# Three‐Dimensional Habitat Structure Drives Avian Functional and Trait Diversity Across North America

**DOI:** 10.1002/ece3.70988

**Published:** 2025-04-23

**Authors:** Colin P. Sweeney, William Peterman, Kaiguang Zhao, Karen Goodell, Benjamin Zuckerberg, Marta A. Jarzyna

**Affiliations:** ^1^ Department of Evolution, Ecology and Organismal Biology The Ohio State University Columbus Ohio USA; ^2^ School of Environment and Natural Resources The Ohio State University Columbus Ohio USA; ^3^ Department of Forest and Wildlife Ecology University of Wisconsin‐Madison Madison Wisconsin USA; ^4^ Translational Data Analytics Institute The Ohio State University Columbus Ohio USA

**Keywords:** 3D habitat structure, avian diversity, functional diversity, habitat composition, habitat configuration, LiDAR

## Abstract

Understanding how three‐dimensional (3D) habitat structure drives biodiversity patterns is key to predicting how habitat alteration and loss will affect species and community‐level patterns in the future. To date, few studies have contrasted the effects of 3D habitat composition with those of 3D habitat configuration on biodiversity, with existing investigations often limited to measures of taxonomic diversity (i.e., species richness). Here, we examined the influence of Light Detecting and Ranging (LiDAR)‐derived 3D habitat structure–both its composition and configuration–on multiple facets of bird diversity. Specifically, we used data from the National Ecological Observatory Network (NEON) to test the associations between 11 measures of 3D habitat structure and avian species richness, functional and trait diversity, and phylogenetic diversity. We found that 3D habitat structure was the most consistent predictor of avian functional and trait diversity, with little to no effect on species richness or phylogenetic diversity. Functional diversity and individual trait characteristics were strongly associated with both 3D habitat composition and configuration, but the magnitude and the direction of the effects varied across the canopy, subcanopy, midstory, and understory vertical strata. Our findings suggest that 3D habitat structure influences avian diversity through its effects on traits. By examining the effects of multiple aspects of habitat structure on multiple facets of avian diversity, we provide a broader framework for future investigations on habitat structure.

## Graphical abstract

We examined how three‐dimensional (3D) habitat structure shapes avian species richness, functional and trait diversity, and phylogenetic diversity. We investigated two components of 3D habitat structure–3D habitat composition, the volume of different niche spaces, and 3D habitat configuration, the arrangement of niche space. Our findings suggest that 3D habitat structure influences avian diversity through its effects on traits, with little to no effect on either species richness or phylogenetic diversity.

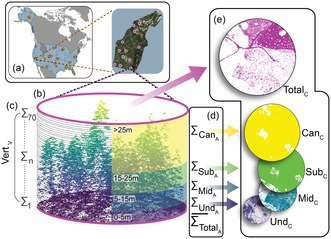

## 1 Introduction

Habitat loss is the leading cause of terrestrial biodiversity declines globally (Pereira et al. 2010, 2012; Jaureguiberry et al. 2022), yet it remains unclear how habitat structure shapes biodiversity (Fletcher et al. 2018; Fahrig et al. 2019). Habitat structure is an umbrella term that encompasses two main concepts: (1) habitat composition, measured as the number or amount of different habitat types and often considered a proxy for niche space and (2) habitat configuration, the spatial arrangement of those habitat types and encompassing multiple axes of spatial heterogeneity (Ben‐Hur and Kadmon 2020; LaRue et al. 2023; Moudrý et al. 2023). Though the number of habitat types typically has a positive and linear (Kerr et al. 2001) or hump‐shaped (Fahrig et al. 2011; Ben‐Hur and Kadmon 2020) relationship with species richness, the effects of habitat configuration on biodiversity remain more ambiguous. Negative, positive, or neutral associations of habitat configuration with biodiversity have all been demonstrated (Valente et al. 2023), variation that might be a result of contextual dependencies of individual studies (Betts et al. 2019; Mimet et al. 2019; Santos et al. 2021; Banks‐Leite et al. 2022) or disparities in the methods used to measure habitat configuration (Fahrig 2017; Fletcher et al. 2018; Fahrig et al. 2019; Valente et al. 2023). Furthermore, though habitat composition and configuration are hypothesized to exert disparate effects on biodiversity (Fischer and Lindenmayer 2007; Villard and Metzger 2014), their impacts are rarely disentangled in practice, potentially leading to inconclusive findings. Finally, and perhaps crucially, habitat structure is predominantly assessed in a two‐dimensional (2D) realm (Ewers and Didham 2008; Haddad et al. 2015), even though the vertical nature of habitat structure has long been recognized to play a crucial role in the assembly and maintenance of communities (MacArthur and MacArthur 1961).

Three‐dimensional (3D) habitat structure incorporates the vertical dimension of habitats alongside the typical horizontal (or 2D measures) of habitat structure. Vertical height strata, such as canopy, subcanopy, midstory, and understory, can be considered different habitat types or niche spaces (Gámez and Harris 2022; Coddington et al. 2023), analogous to different landcover types in a 2D realm. Thus, 3D habitat composition measures the volume of such height strata, analogous to total habitat amount in a 2D context. 3D habitat configuration, on the other hand, considers the horizontal arrangement of vegetation, similar to 2D habitat configuration, but also incorporates the vertical arrangement of vegetation, both within and between different height strata.

Though 3D habitat structure is a relatively new term, it has long been thought to be particularly important for volant taxonomic groups such as birds (MacArthur and MacArthur 1961; Robinson and Holmes 1982; Davies and Asner 2014), likely influencing large scale patterns of avian diversity. While positive associations between avian species richness and vegetation at various height strata have been previously reported (Lesak et al. 2011; Vogeler et al. 2014; Zellweger et al. 2017; Feng et al. 2020; Burns et al. 2020), many ascribe this relationship to an increase in available niche space, without explicitly examining the effects of 3D composition versus 3D configuration (Richardson and Moskal 2011). The few studies that have investigated both 3D habitat composition and configuration present conflicting findings. While some studies find a greater effect of 3D habitat composition on avian richness compared to habitat configuration (Lesak et al. 2011; Vogeler et al. 2014), others find 3D habitat configuration to be a more important driver of bird richness, with both positive (Goetz et al. 2007) and negative (Carrasco et al. 2019) effects reported. These inconsistencies suggest that our understanding of the effects of 3D habitat structure on avian diversity is incomplete, ultimately hampering any attempts to elucidate the mechanisms underlying vegetation structure‐biodiversity associations.

Inconsistencies in the reported effects of 3D habitat structure on biodiversity may in part stem from the exclusion of relevant dimensions of biodiversity from studies of vegetation‐biodiversity associations. For example, while habitat composition consistently emerges as the primary predictor of avian diversity, the effects of 3D habitat configuration varies across different functional groups (Davison et al. 2023). This pattern suggests that the link between habitat structure and biodiversity is likely mediated by species traits (Valente and Betts 2019; Jones et al. 2023), which is further supported by the documented associations between habitat heterogeneity and traits such as foraging stratum, diet, body mass, beak shape, and hand‐wing index (HWI) (Weisberg et al. 2014; Stirnemann et al. 2015; Coddington et al. 2023). 3D habitat structure might thus directly influence trait composition of an assemblage. As a result, biodiversity measures that capture the range of functional trait values within an assemblage, such as functional diversity (FD) (Petchey and Gaston 2002), should correlate more strongly with habitat structure than trait‐agnostic measures like species richness. Specifically, we expect that different height strata represent distinct microhabitats and resource niches that only species with particular traits can exploit (Gámez and Harris 2022). Thus, the addition and/or loss of a particular height strata(s) should represent an addition or loss of a unique niche(s) and a change to FD. Furthermore, increased heterogeneity within and between height strata should filter for traits like dispersal ability and body size (Claramunt et al. 2022), making 3D configuration also likely to impact FD. Consequentially, we theorize that measures of FD should be more responsive to changes in 3D habitat structure compared to TD, given that individual traits that comprise FD should occur based on the presence and arrangement of a particular niche while TD should respond primarily to the overall size of niche space (Pellissier et al. 2018).

Phylogenetic diversity (PD), that is, the diversity of evolutionary histories within an assemblage (Faith 1992), is shown to correlate with some measures of habitat structure (Rurangwa et al. 2022), but is understudied when it comes to measures of 3D habitat structure. Given that closely related species often have conserved morphological and behavioral traits (Wiens and Graham 2005, Gianuca et al. 2014; but see E‐Vojtkó et al. 2023), 3D habitat structure likely influences PD through environmental habitat filtering (Weiher and Keddy 1999) or phylogenetic habitat filtering (Duarte 2011). Habitats with higher levels of 3D habitat composition should provide more opportunities for species specialization and result in higher PD, while habitats with lower 3D habitat composition should have more acute environmental filtering and lead to lower and more clustered PD (Gianuca et al. 2014). More research is needed, however, to understand the relationship between avian PD and metrics of both 3D habitat composition and configuration.

Here, we leverage high‐resolution Light Detecting and Ranging (LiDAR) (Bergen et al. 2009) and avian survey data from the National Ecological Observatory Network (NEON) (Hargrove and Hoffman 1999), paired with trait and phylogenetic information to investigate the associations of 3D habitat structure with avian species richness, trait and functional diversity, and phylogenetic diversity across North America. We test the effects of a comprehensive suite of measures of 3D habitat composition and 3D habitat configuration, allowing us to gain deeper insights into the mechanisms of avian community assembly along vegetation gradients. We hypothesize that 3D habitat composition will show more consistent relationships with avian diversity across all diversity dimensions compared to 3D habitat configuration, owing to its association with available niche space. We also hypothesize that functional diversity and individual traits will respond stronger to 3D habitat structure compared to either species richness or phylogenetic diversity, since traits and trait‐derived metrics should follow shifts in niche space.

## 2 Methods

### 2.1 Data

*Avian data*. To conduct our study, we used National Ecological Observatory Network's breeding landbird point count dataset (data product: DP1.10003.001). We chose to use NEON data despite its lack of resolution for recording individual birds within specific vegetation strata, as it is one of the few datasets that collects both avian and high‐resolution LiDAR data across a large spatial extent and in a standardized way. Indeed, studies of 3D habitat structure often pair census data with 3D vegetation structure to infer relationships between the two, even when data for the vertical positions of birds are unavailable (MacArthur and MacArthur 1961; Carrasco et al. 2019). We used data from 2017, as it was the year with the greatest number of avian survey plots available. NEON carries out point count surveys for breeding landbirds at each of its 47 terrestrial sites, with sites located across 20 North American ecological “Domains” (Kao et al. 2012). Each NEON site uses one of two avian survey methodologies. Larger NEON sites use gridded plots with each of the nine survey points spaced 250 m apart in a 3 × 3 grid and are sampled once during the breeding season. Smaller NEON sites use single‐point plots and are sampled twice during the breeding period. Each site has a variable number of bird plots (5–10 for sites with gridded plots and 5–25 for sites with single‐point plots). At each point within a given plot, observers record the total number of birds seen or heard within a 6‐min time period, making note of the distance to each individual bird and plot‐level environmental metrics (Thibault 2018). For gridded plots, 6‐min surveys are conducted in each of the nine survey points sequentially, with each point meant to represent a spatial replicate akin to the temporal replicates of the single‐point plots (Pavlacky et al. 2012; Thibault 2018). Surveys are timed to coincide with the breeding season at each NEON site, to best capture the abundance of resident breeding birds (Table S1) (Kao et al. 2012).

**FIGURE 1:**
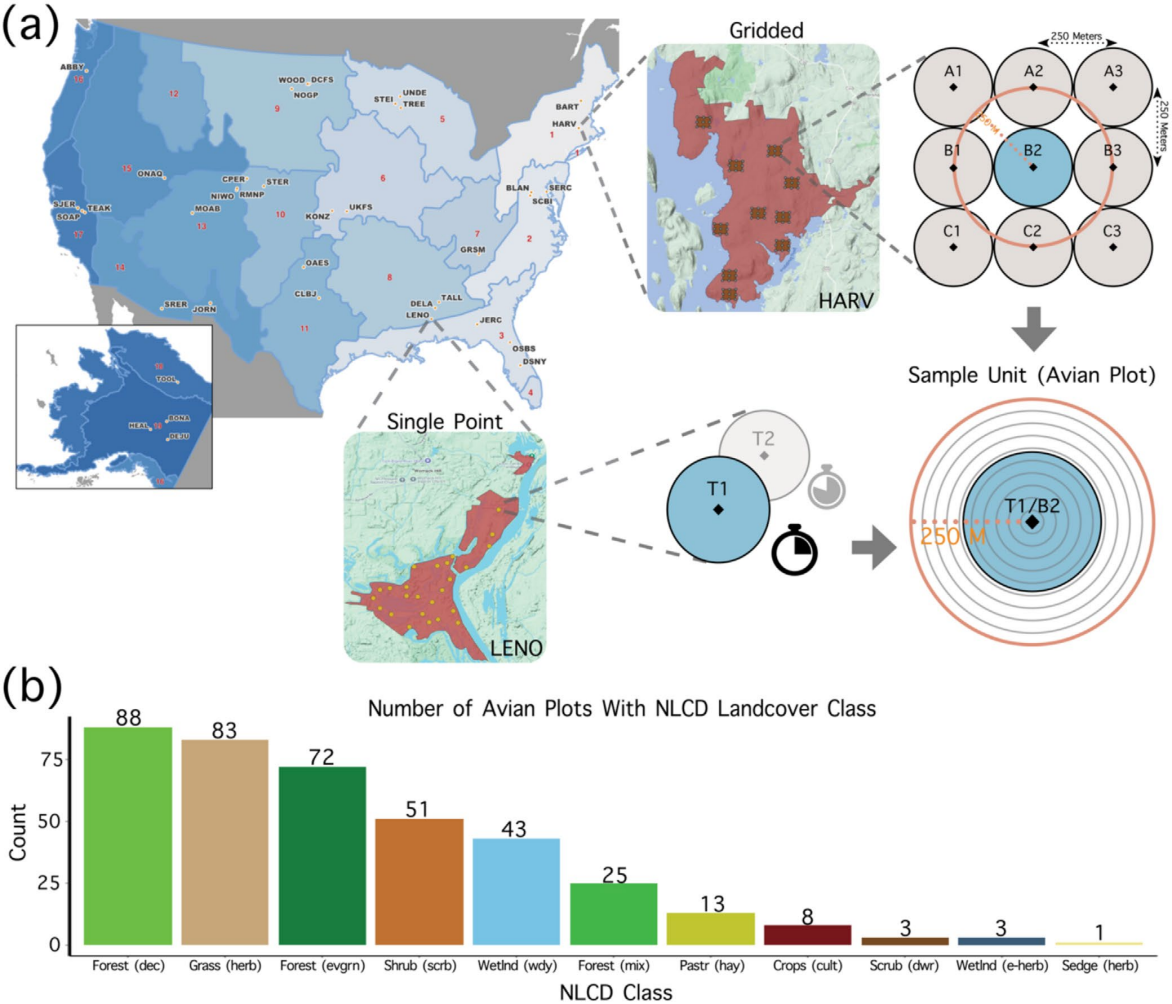
(a) Subsampling of NEON avian plot data. The study utilized data from 38 NEON sites, each implementing either gridded avian plots (e.g., HARV) or single‐point avian plots (e.g., LENO). Gridded plots consist of nine survey points spaced 250 m apart; for analysis, only the central point (B2) was retained. Single‐point plots are sampled twice per breeding season, with only the first sample period (T1) retained for analysis. Upon filtering avian sampling units, we used a 250 m buffer from the center of each plot to derive LiDAR structure metrics (indicated in orange) and 10 m distance bins for the distance sampling model (depicted as gray concentric circles). (b) Distribution of land cover types at avian plots across all NEON sites included in the study. Land cover classifications follow National Land Cover Data (NLCD) categories and were obtained from NEON data: “Forest (dec)” = “deciduousForest”, “Grass (herb)” = “grasslandHerbaceous”, “Forest (evgrn)” = “evergreenForest”, “Shrub (scrb)” = “shrubScrub”, “Wetlnd (wdy)” = “woodyWetlands”, “Forest (mix)” = “mixedForest”, “Pastr (hay)” = “pastureHay”, “Crops (cult)” = “cultivatedCrops”, “Scrub (dwr)” = “dwarfScrub”, “Wetlnd (e‐herb)” = “emergentHerbaceousWetlands”, “Sedge (herb)” = “sedgeHerbaceous”.

While both gridded plots and single‐point plots are designed to estimate detection probability through occupancy modeling, the spatial replication used in gridded plots operates under different closure assumptions than temporal replication used in single‐point plots (Rota et al. 2009; Pavlacky et al. 2012). In both methods, it is assumed that individuals remain within the plot during sampling; however, spatial replicates are limited to a single morning, while temporal replicates occur over the span of a month. For this reason, we did not think it appropriate to treat these two forms of replication as equivalent. Furthermore, both survey methodologies use different levels of sampling effort per plot, with gridded plots having four and a half times the sample effort as single plot sites. To standardize these data and allow for both single‐point plots and gridded plots in our analysis, we retained only the central point at NEON sites with gridded plots and the first sampling period at NEON sites with single‐point plots (see Figure 1). We further retained only those plots that overlapped with NEON Airborne Observation Platform (AOP) LiDAR data (see below) collected in 2017. In total, we used 385 avian plots from 38 NEON terrestrial sites across 17 of the 20 NEON Domains. Vegetation at avian plots ranged from scrubland to forest in terms of height, with roughly 50% of plots designated as forest according to NEON's own landcover classifications (see Figure 1 for full list of vegetation types). As avian sampling occurred during the daytime, we excluded all nocturnal species from all avian plot counts, resulting in a total of 260 species in this analysis (see Dryad data repository for full list of species).

*Other biodiversity data*. To calculate FD and PD, we took advantage of a number of existing datasets. We used trait data from the AVONET (Tobias et al. 2022) and EltonTraits 1.0 databases (Wilman et al. 2014). We used five trait categories: diet, foraging stratum, and body mass from Wilman et al. (2014) and beak measurements and hand‐wing index (HWI) from Tobias et al. (2022). The diet category included 10 axes: proportion of diet that is insects, amphibians and reptiles, mammals and birds, fish, unknown vertebrates, carrion, nectar, fruit, seeds, and other plant matter. The foraging stratum category included seven axes: proportional use of aerial, canopy, mid‐height, understory, ground, water above surface, and water below surface. The beak category included three axes: beak width, beak depth, and beak length. The categories of body mass and HWI both had a single trait axis. We followed the latest guidelines regarding trait selection (Schleuter et al. 2010; de Bello, Carmona, et al. 2021) and retained the original trait axes within each trait category to preserve the highest resolution of trait information available, rather than collapsing trait axes into binary traits (e.g., invertivore or not, frugivore or not). To calculate PD, we obtained a random sampling of 100 phylogenetic trees from the Bird Tree database, using the Ericson backbone (Jetz et al. 2012).

*LiDAR data*. To quantify 3D habitat structure, we used NEON's AOP LiDAR data, which are flown above NEON sites during peak vegetation greenness and have a minimum density of 4 LiDAR shots per square meter (data product: DP1.30003.001) (Kampe et al. 2010). We downloaded all LiDAR point cloud data that overlapped with each of the 385 plots from the NEON repository from 2007 using the R package “neonUtilities” (Lunch et al. 2024).

*Environmental data*. We also collected environmental data to control for factors known to drive avian diversity across large spatial gradients. Elevation and latitude of each avian plot were taken directly from the NEON database while climatic data were obtained from Daymet, which produces interpolated estimates of daily weather at a 1 km × 1 km gridded spatial resolution (Thornton et al. 2022). We selected temperature range (i.e., min‐max) of the NEON avian survey period (May–June) as our sole climatic variable after first testing multiple candidate climatic variables and removing those highly correlated with elevation and/or latitude. Temperature range was taken at the centroid of each avian plot using the R package “daymetr” (Hufkens et al. 2018).

### 2.2 Species Abundance

We corrected for imperfect detection in avian count data using a Bayesian Hierarchical Distance Sampling (BHDS) model (Buckland et al. 2001; Oedekoven et al. 2014). The BHDS model consisted of a hierarchically linked ecological process model (Equation 1) and detection model (Equation 2). The ecological process model related environmental covariates (elevation and latitude) to species abundance via a Poisson model (Equation 1), where the indices j and s refer to individual sites and species, $\lambda_{\mathrm{js}}$ is the term connecting the log‐link function to the mixed effect model, β's are the model coefficients, and N is the estimated abundance.

(1) $$\log\left(\lambda_{\mathrm{js}}\right)=\beta_{0}+\beta_{1s}\times {\text{elevation}}_{j}+\beta_{2s}\times {\text{latitude}}_{j}$$

$$N_{\mathrm{js}}\sim \text{Poisson}\left(\lambda_{\mathrm{js}}\right)$$

The detection model (Equation 2) was used to model declining rates of detection with increasing distance from the observer using a half normal detection function (Buckland et al. 2001). We chose a half normal detection function following a similar formulation used by Kéry and Royle (2016) and Sollmann et al. (2016). For computational simplicity, we used 10—25‐m wide distance bins in the detection model; distance bins have been shown to yield similar estimates to continuous distance formulations given fine enough distance bin resolution (Buckland et al. 2001). Detection probability was a function of cloud cover, air temperature, windspeed, and observer identity: where the indices k, s, and j refer to distance bin, species, and site, respectively; $\sigma_{\mathrm{sj}}$ is the term connecting the log‐link function to the half normal detection function (i.e., $g\left(x;\theta \right)=\text{exp}\;\left(-\frac{x^{2}}{2\sigma^{2}}\right)$); α's are model coefficients; $p_{\mathrm{sk}}$ is the probability of detection (specified here as a half normal detection function); $\delta_{k}$ is the distance to the observer, and $C_{\mathrm{js}}$ is observed count.

(2) $$\left(\sigma_{\mathrm{sj}}\right)=\alpha_{0}+\alpha_{1}\times {\text{cloudcover}}_{s}+\alpha_{2}\times {\text{airtemp}}_{s}+\alpha_{3}∙{\text{wind}}_{s}+\alpha_{3}\times {\text{observer}}_{s}$$

$$p_{\mathrm{sk}}=\exp\left(-\delta_{k}\times \frac{\left(\delta_{k}\right)}{\left(2\times \sigma_{s}^{2}\right)}\right)$$

$$C_{\mathrm{js}}\sim \text{Binomial}\left(N_{\mathrm{js}}p_{\mathrm{sk}}\right)$$

We used both species‐level parameters and community‐level hyper parameters, which allowed us to better estimate abundances ($\hat{N}$) for rare species for a given area (Sollmann et al. 2016). All BHDS model calculations were run using the R package “jagsUI” (Plummer 2003; Kellner 2015) following model formulation similar to those outlined in Kéry and Royle (2016) and Sollmann et al. (2016). To enable comparability among species and locations, $\hat{N}$ of each species was calculated using a circular plot of 250 m radius. To avoid instances where the BHDS model estimated $\hat{N}>0$ for species that are unlikely to be present given their ecological constraints, we *post hoc* filtered detection corrected species abundance estimates so that only species whose geographic ranges overlapped a NEON Domain within which a given plot was embedded could have $\hat{N}\geq 1$ at that plot. Expert range maps used for filtering were obtained from BirdLife International (2022). Lastly, species whose $\hat{N}<0.95$ at a given plot were considered to be absent at that location, that is their $\hat{N}$ was fixed to 0, while those whose $\hat{N}\geq 0.95$ were considered to be probable presences and retained for further analysis. The threshold of 0.95 rather than 1 was used to ensure inclusion of species with highly likely presences. The resulting species $\hat{N}$ were used as inputs for quantification of avian species richness, functional and trait, and phylogenetic diversity.

Note that we chose to exclude 3D habitat variables from both the ecological and detection equations of the BHDS model because we thought it unlikely for all species to exhibit similar responses to the same habitat structure metrics. Thus, including 3D habitat variables could cause the community‐level hyper‐parameters to produce less realistic abundance estimates for individual species.

### 2.3 Avian Diversity

Avian species richness (SpRich) was calculated for each plot as the sum of all species whose abundance estimates $\hat{N}\geq 0.95$, again using $\hat{N}$ = 0.95 as the threshold to ensure the inclusion of probable presences.

We calculated functional diversity using three complimentary but independent functional components: Functional richness (FRich), functional evenness (FEven), and functional divergence (FDiv) (Villéger et al. 2008). FRich measures the breadth of the trait space occupied by a given assemblage, FEven is a measure of regularity of the distribution of species abundances in trait space, and FDiv measures the proportion of the trait space represented by extreme trait values (Villéger et al. 2008; Mouillot et al. 2013). To obtain these metrics, we first calculated multivariate trait dissimilarity among all species found in all plots using a corrected Gower's distance (Gower 1971; de Bello, Botta‐Dukát, et al. 2021). A corrected Gower's distance better balances the contribution of trait categories and axes to overall dissimilarity (de Bello, Botta‐Dukát, et al. 2021) and can handle quantitative, semi‐quantitative, and qualitative traits (Botta‐Dukát 2005). We designated diet, foraging niche, body mass, beak measurements, and hand‐wing index as distinct trait categories, and optimized traits weights for each category and their axes using 300 iterations of the optimization algorithm from the “gawdis” package (de Bello, Botta‐Dukát, et al. 2021). The resulting multivariate trait dissimilarity matrix was then fed into the function “dbFD” in the R package “FD”, along with a plot‐level abundance matrix, to quantify FRich, FEven, and FDiv (Laliberté et al. 2023). FRich was quantified as a minimum convex polygon, FEven was quantified as the minimum spanning distance between all species in the trait space, and FDiv was quantified as the average distance of all species to the centroid of the trait space.

FRich is closely correlated with species richness (Cornwell et al. 2006). To correct for this association, we generated a null model expectation for each NEON plot. We first reshuffled (100 times) species abundance estimates using random independent swaps between plots within each NEON Domain, while keeping plot‐level species richness constant. For each NEON plot, only species whose range boundaries overlapped with the NEON Domain of that plot were allowed to “appear” in that assemblage. For each of the 100 null communities, we calculated the resulting null expectation of FRich. Finally, we calculated the standardized effect sizes (SES) of the deviation of the observed values of FRich from those expected given species richness as $\mathrm{SES}=\frac{\left(\mu_{\mathrm{obs}}-\mu_{\text{null}}\right)}{{\mathrm{sd}}_{\text{null}}}$, with μ representing mean values and sd representing standard deviation. SES were considered species richness‐corrected values of FRich (FRich_SES_) and used in all subsequent analyses. FEven and FDiv are abundance‐weighted and not related to species richness, and thus not requiring a correction (Laliberte and Legendre 2010).

We additionally quantified the prevalence of different trait characteristics in each assemblage. First, using the Gower's distance matrix for each pairwise combination of species, we ran a Principal Coordinate Analysis (PCoA) using the “cmdscale” function in the “stats” R package. We assessed the quality of the functional space by evaluating the congruence between the distances of species pairs and the initial functional distances, using the square root of the mean squared deviation (RMSD) metric (Maire et al. 2015; Magneville et al. 2022). Diagnostic plots were run using the “mFD” package's “quality.fspaces.plot” function (Magneville et al. 2022). Based on RMSD values and diagnostic plots (see Figure S1), we observed a substantial improvement in congruence when using four PCoA aces, while axes five and size contributed only marginally; we thus retained the first four principal coordinates (PC) axes for further analysis. PC scores associated with each species, weighted by their plot‐level abundance estimates $\hat{N}$, were then averaged at the plot level to obtain assemblage‐level estimates for each PC axis. To determine how individual traits were correlated with each PC axis, trait vectors were fit to the PCoA ordination using the “vegan” package's “envfit” function (Oksanen et al. 2022).

Finally, for each NEON plot, we quantified phylogenetic diversity using two indices, Mean Pairwise Distance (MPD) and Faith's Phylogenetic Diversity (FPD) using the R package “picante” (Kembel et al. 2010). MPD and FPD were based on the distribution of 100 phylogenetic trees from Bird Tree (Jetz et al. 2012). MPD quantifies the average phylogenetic tree branch length between the closest pairs of relatives in a given assemblage. FPD is the sum of all phylogenetic branch lengths for a given assemblage (Tucker et al. 2017). As both MPD and FPD are correlated with species richness, we then corrected both indices by comparing each index against a null distribution of values, which we generated using the same 100 null communities used to generate FRich_SES_ and the same randomization procedure. We created null estimates for each of the 100 phylogenetic trees, resulting in 10,000 total “null” trees. We then quantified SES, which we considered species richness‐corrected values of MPD (MPD_SES_) and FPD (FPD_SES_).

### 2.4 3D Habitat Structure

We pre‐processed LiDAR data by first clipping NEON pointcloud data using a 250 m radius circle measured from the centroid of each of the 385 selected avian plots. Raw pointcloud data was then height normalized to eliminate height differences due to topographic variation using the function “normalize_height” from the “lidR” package (Roussel et al. 2020) and a digital terrain model (DTM) generated with a k‐nearest neighbor approach with an inverse‐distance weighting using the function “knnidw” and default settings. To eliminate outlier values, LiDAR points that fell below zero height after height normalization or those high above the vegetation were removed using function “filter_poi” from “lidR” package.

**FIGURE 2:**
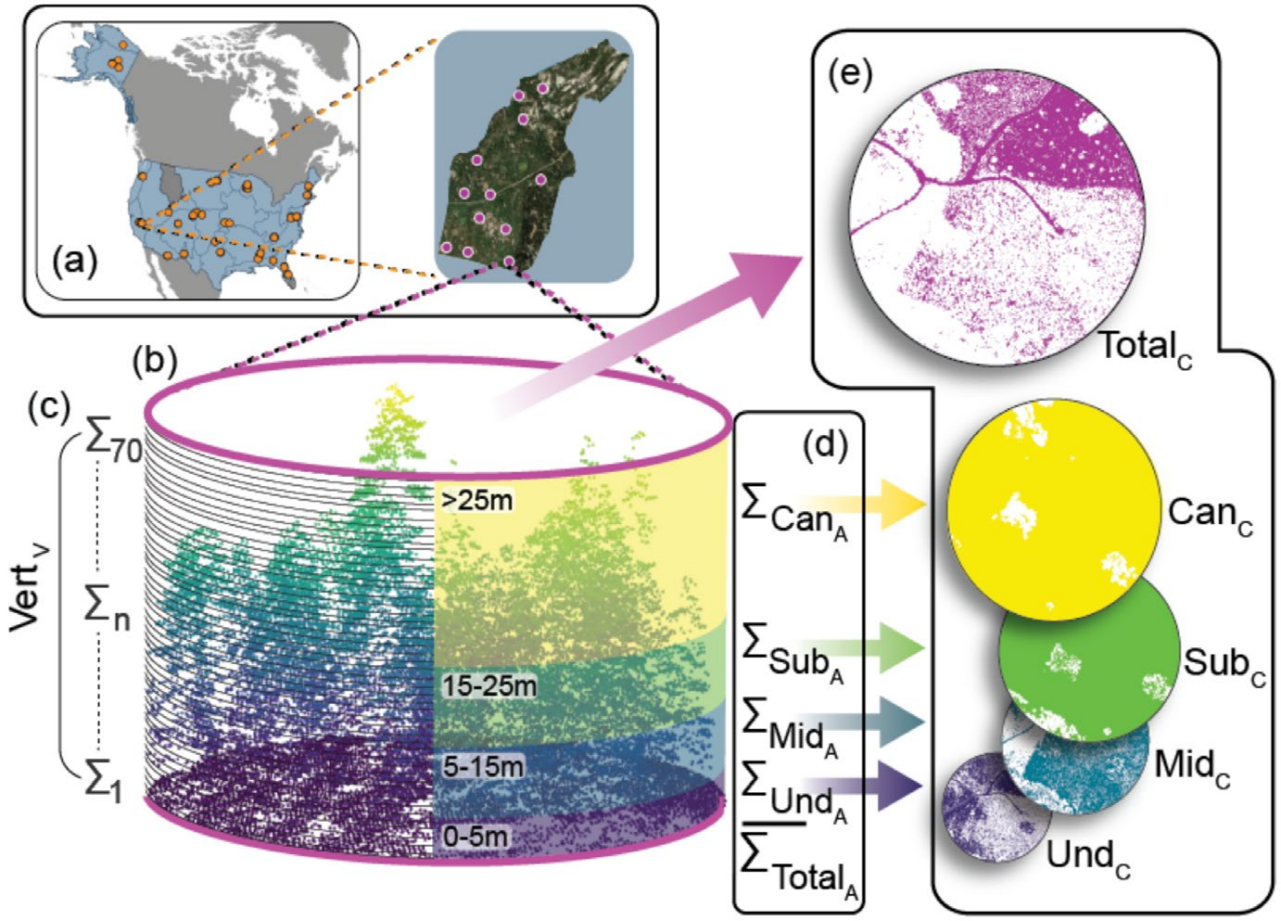
Conceptual Diagram of 3D habitat structure calculations. For each of the 385 NEON plots we studied (a), LiDAR data were split into 1 m bins (b, left) and discretized into four height strata (understory, Und, 0‐5 m; midstory, Mid, 5‐15 m; subcanopy, Sub, 15‐25 m; canopy, Can, > 25 m; b, right). Vertical habitat configuration was quantified as the variance of number of voxels found in each 1 m height bin (Vert_V_; c). 3D habitat composition was quantified as the number of voxels at each height bin (Und_A_, Mid_A_, Sub_A_, Can_A_) and the sum of all voxels (Total_A_; d). 3D habitat configuration at each height stratum (Und_C_, Mid_C_, Sub_C_, Can_C_) was quantified by converting the voxels in each height stratum into rasters before quantifying the number of habitat patches in the raster and 2D habitat configuration (Total_C_) was quantified by converting all voxels in the plot into a raster and then quantifying the number of habitat patches (e).

We derived a suite of 3D habitat compositional and configurational metrics using 0.5 m x 0.5 m x 0.5 m voxels (3D‐rasters), which were obtained by converting pointclouds into voxels using the function “voxelize_points” from package “lidR” (Roussel et al. 2020). To obtain strata‐specific measures of habitat structure, voxels were split into four vertical height bins: understory (0–5 m), Und; midstory (5‐15 m) Mid; subcanopy (15–25 m) Sub; and canopy (> 25 m) Can (Figure 2b). Note that while we used nomenclature for the four height strata that is consistent with nomenclature in forest‐related disciplines, the selection of strata thresholds were based on those commonly used in ecological studies (Whitehurst et al. 2013; Coddington et al. 2023).

We calculated 10 measures of 3D habitat structure (five measures of 3D habitat composition and five measures of 3D habitat configuration) and one measure of 2D habitat structure for comparison. 3D habitat composition was quantified as total habitat amount (Total_A_) and as habitat amount within each of the four vertical strata. Total_A_ was calculated by summing the total number of voxels found along the entire vegetation profile within the 250 m radius (Figure 2d). Habitat amounts of each vertical stratum (understory, Und_A_; midstory, Mid_A_; subcanopy, Sub_A_; canopy, Can_A_) were calculated as the sum of all voxels within each stratum for each 250 m radius plot (Figure 2d). We quantified 3D habitat configuration as vertical habitat configuration along the entire vegetation profile and as habitat configuration at each of the four vertical strata. To quantify vertical habitat configuration, we first assigned each voxel into one of seventy 1 m vertical height bins and summed the total number of voxels in each bin. We then quantified vertical habitat configuration of the resulting array as the variance of that array (Vert_V_) (Figure 2g). Note that though Shannon's Diversity Index (SDI) is often used to quantify vertical habitat configuration (Carrasco et al. 2019), we opted to not use SDI in our analysis because of its high correlation with total habitat amount in our study system. To obtain strata‐specific measures of habitat configuration (Und_C_, Mid_C_, Sub_C_, Can_C_), we used voxels associated with each vertical stratum and converted them into 2D raster layers (Petras et al. 2017) (Figure 2e). For each strata‐specific raster layer, we then calculated habitat configuration as the number of patches (NP) (Fahrig 2003), using the R package “landscapemetrics” (Hesselbarth et al. 2019). We selected NP as our metric of habitat configuration as it can be independent of habitat amount (Neel et al. 2004; Wang et al. 2014) and is often used in 2D fragmentation studies—with high numbers of patches representing high levels of heterogeneity/patchiness and low numbers of patches representing low levels of heterogeneity/patchiness. Finally, we quantified a total 2D habitat configuration (Total_C_) by converting Total_A_ into a single 2D raster and calculating NP (Figure 2e).

### 2.5 Bayesian Mixed Effect Models

To understand the influence of 3D and 2D habitat structure on avian diversity, we fitted 12 hierarchical Bayesian mixed effect models to each metric of avian diversity: SpRich, FD (FRich_SES_, FEven, FDiv), traits (PC1, PC2, PC3, PC4), and PD (FPD_SES_, MPD_SES_). All models followed the same general formulation (Equation 3), where y is the response variable, α is the intercept, and β terms are slope (coefficient) estimates for each covariate. To control for random site effects, we fitted NEON site IDs as random intercepts. As NEON sites are hierarchically arranged within district ecological Domains (see Figure 1) (Kampe et al. 2010), including NEON site IDs as random effects also helps account for some of the inherent ecological variation among avian plots. Models were fitted with a Poisson (SpRich), Beta (FEven), or Gaussian (all others) distributions.

(3) $$\text{Biodiversity}∼\text{Distribution}\left(y_{i}\right);Ϝ=\text{link function}$$

$$Ϝ\left(y_{i}\right)=\alpha +\beta 1\times {\text{covariate}}_{1}+\ldots \mathrm{\beta n}\times {\text{covariate}}_{N}+\left(1|\text{site}\right)$$

We chose to apply a model selection approach, wherein each of the 12 models represented a different hypothesis and thus comprised different combinations of habitat and environmental covariates (Table 1). To avoid multicollinearity, we considered a Pearson correlation coefficient of 0.7 or higher to be problematic (Dormann et al. 2013) and removed any candidate variables that were correlated at or above this threshold. Total_A_, however, whose correlation with both Mid_A_ and Sub_A_ exceeded 0.7 (Figure S2), was considered too important of a variable to fully exclude from our analysis. We therefore ran two versions of the full model, with and without Total_A_, and opted to proceed with the version without Total_A_ for all further statistical inferences. This decision was based on the poor fit of the full model that included Total_A_. Note that while running univariate models or models using orthogonal environmental axes derived from a PCA could have also addressed the issue of multicollinearity, these approaches would not have aligned with our hypotheses and would have hindered our ability to compare the effect sizes of different environmental covariates. Additionally, while we did include elevation and latitude as covariates in both the ecological equation of the BHDS model and the main mixed effect models, such an approach is unlikely to greatly impact parameter estimates in the mixed effect models. Estimates of variance for elevation and latitude use different dependent variables in the BHDS models and mixed effect models and thus have different underlying statistical distributions that are independent from one another.

**TABLE 1:** Model specification for the 12 Bayesian mixed effect models relating avian diversity to habitat structure and environment covariates. All 12 models were used to model the 10 biodiversity indices. Models contained different combinations of compositional habitat structure (Total_A_, Can_A_, Sub_A_, Mid_A_, Und_A_), configurational habitat structure (Total_C_, Can_C_, Sub_C_, Mid_C_, Und_C_, Vert_V_), and environmental variables (TempRange, Elevation, Latitude). Color designates parameters in broad categories of environmental (pink), habitat composition (blue), and habitat configuration (orange).

Model Name	Model description	Independent variables
(1). Full	Contains all structural and environmental variables	TempRange+ Elevation + Latitude + Total_A_ + Can_A_ + Sub_A_ + Mid_A_ + Und_A_ + Total_C_ + Can_C_ + Sub_C_ + Mid_C_ + Und_C_ + Vert_V_
(2). Full_minusTotVol	Contains all structural and environmental variables except TotalA	TempRange+ Elevation + Latitude + Can_A_ + Sub_A_ + Mid_A_ + Und_A_ + Total_C_ + Can_C_ + Sub_C_ + Mid_C_ + Und_C_ + Vert_V_
(3). Structure	Contains all structural variables but not environmental variables	Can_A_ + Sub_A_ + Mid_A_ + Und_A_ + Total_C_ + Can_C_ + Sub_C_ + Mid_C_ + Und_C_ + Vert_V_
(4). Configuration	Contains only configurational variables	Total_C_ + Can_C_ + Sub_C_ + Mid_C_ + Und_C_ + Variance
(5). Configuration_env	Contains configurational variables and environmental variables	TempRange+ Elevation + Latitude + Total_C_ + Can_C_ + Sub_C_ + Mid_C_ + Und_C_ + Vert_V_
(6). Composition	Contains only compositional variables	Can_A_ + Sub_A_ + Mid_A_ + Und_A_
(7). Composition_env	Contains compositional variables and environmental variables	TempRange+ Elevation + Latitude + Can_A_ + Sub_A_ + Mid_A_ + Und_A_
(8). Horizontal	Contains all structural variables except for vertical variance	Can_A_ + Sub_A_ + Mid_A_ + Und_A_ + Can_C_ + Sub_C_ + Mid_C_ + Und_C_
(9). Horizontal_env	Contains all structural variables except for vertical variance and environmental variables	TempRange+ Elevation + Latitude + Can_A_ + Sub_A_ + Mid_A_ + Und_A_ + Can_C_ + Sub_C_ + Mid_C_ + Und_C_
(10). Amount	Contains only TotalA and environmental variables	TempRange+ Elevation + Latitude + Total_A_
(11). Vertical	Contains TotalA and vertical variance and environmental variables	TempRange+ Elevation + Latitude + Total_A_ + Vert_V_
(12). Climate	Environmental variables only	TempRange+ Elevation + Latitude

We ran all models using the R package “brms” (Bürkner 2017). All covariates were centered and scaled prior to the analysis. Prior and posterior predictive checks were conducted and we evaluated models for MCMC convergence (Kruschke 2021). We used leave‐one‐out (LOO) comparisons from the “loo” R package (Vehtari et al. 2017; Kruschke 2021) to compare the relative fit of all models. Following existing practice (Sivula et al. 2022), models were considered significantly different from one another when the pairwise difference in expected log pointwise predictive density was at least three times the standard error (Table S2). In cases where no one model was chosen as top performing, all equally top performing models were used to inform overall covariates' coefficient estimates and their effect sizes.

## 3 Results

### 3.1 Trait Space

**FIGURE 3:**
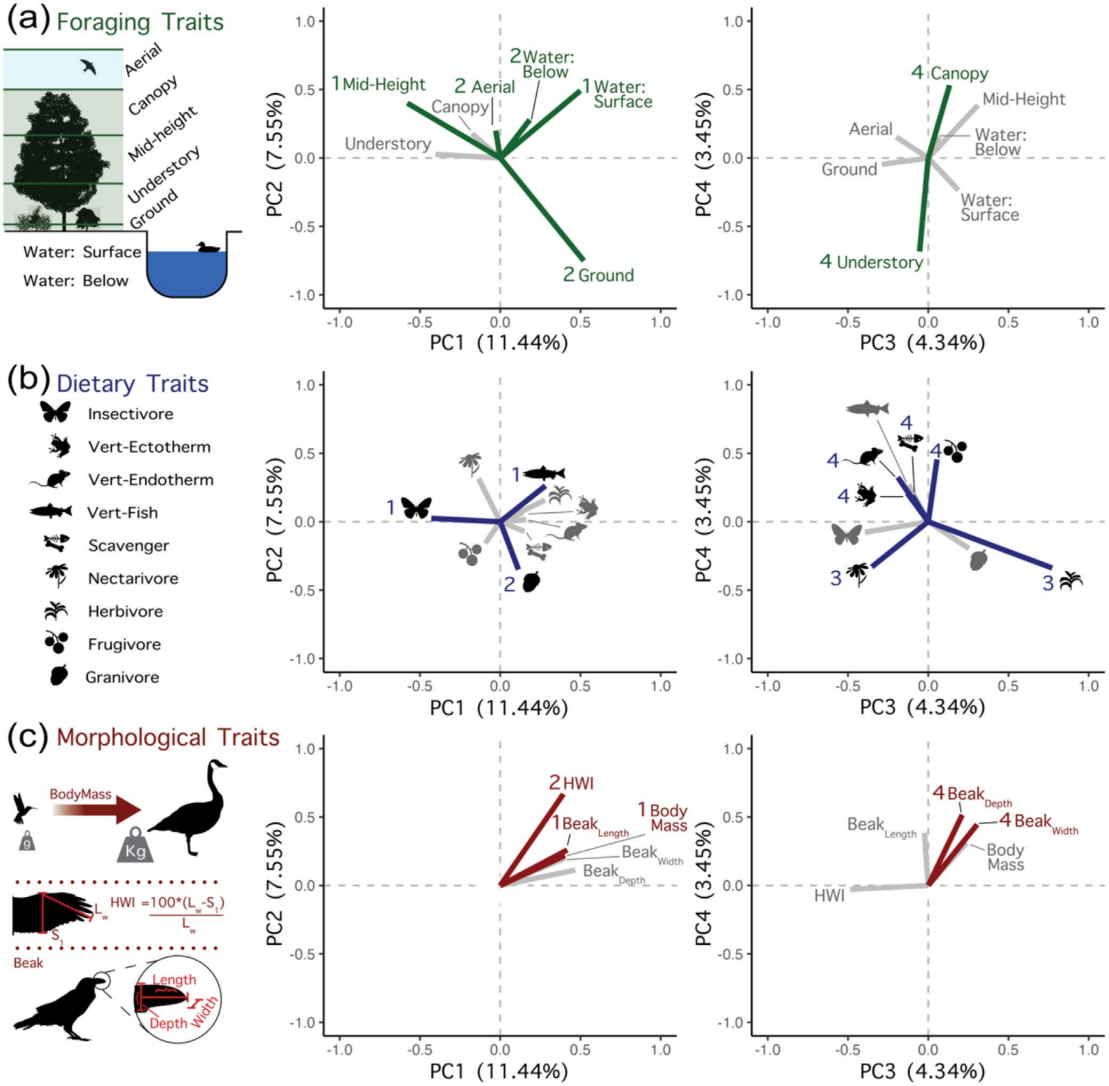
Vectors from Principal Coordinate Analysis (PCoA) for the first four Principal Coordinates (PCs) for foraging (a), dietary (b), and morphological (c) traits. The numbers associated with each trait loading specify the PC axis upon which the respective trait exhibits the strongest loading. Vectors are shaded in gray for traits that do not display the strongest loading on the given PC axis. Unknown vertebrate was left off of dietary trait plots because no species in our analysis had this trait axis.

Water‐surface foraging, piscivorous diet, body mass, and beak length loaded positively on PC1, while insectivorous diet and mid‐canopy foragers loading negatively on PC1. Aerial and below‐water foraging and HWI loaded positively on PC2, while granivore diet and ground foraging loaded negatively on PC2. Herbivorous diet loaded positively on PC3, while nectarivorous diet loading negatively on PC3. Lastly, canopy foraging, ectotherm diet, endotherm diet, scavenger diet, frugivorous diet, beak depth, and beak width loaded positively on PC4, while understory foraging loading negatively (Figure 3, and Table S3).

**FIGURE 4:**
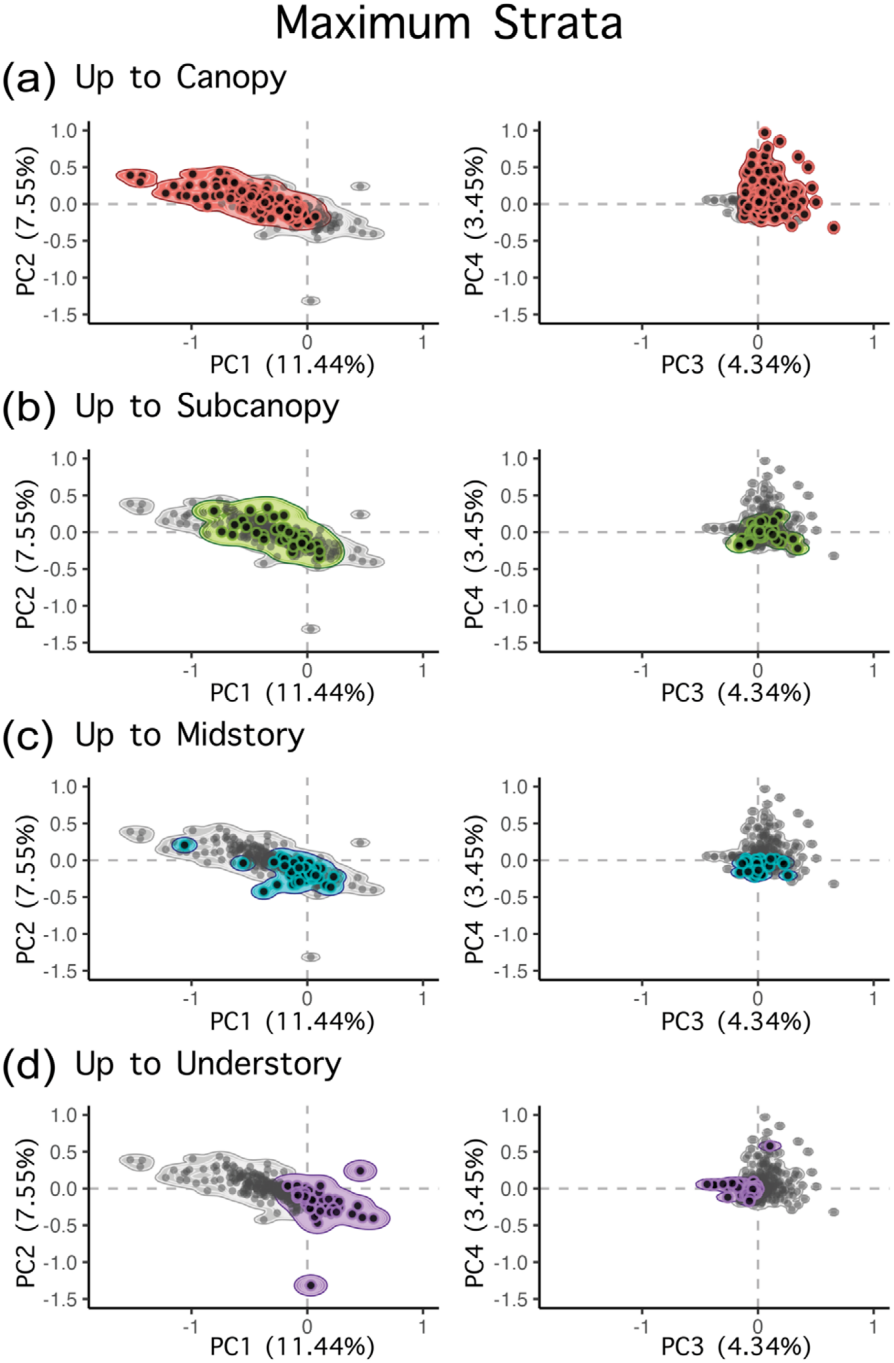
Principal Coordinate (PC) trait space for all avian plots (gray) and avian plots subsetted based on the maximum height strata occupied by vegetation (color): Canopy (a), Subcanopy (b), Midstory (c), and Understory (d). Color indicates plots with vegetation that reaches but does not exceed the four height strata. Points indicate NEON plots; values for PC1—PC4 for each NEON plot represent average PC scores for all species occurring at that plot, weighted by their abundance estimates. All density plots are generated with contour lines up to the 100% percentile.

We found a shift in trait space along the vertical profile of the vegetation (Figure 4). Bird assemblages in plots with vegetation up to the canopy height stratum and, to a lesser extent, up to subcanopy height stratum occupied larger trait volumes than assemblages in plots with vegetation up to only midstory or understory height stratum (Figure 4). Trait space also shifted along the PC axes from low to high vertical height strata, reflecting a shift from predominantly understory and ground foraging birds (negative PC2 and PC4) to canopy and midstory foraging birds (positive PC2 and PC4). Additionally, plots with only understory vegetation tended to harbor communities comprised of species with on average larger bodies and aquatic lifestyles (positive PC1), in contrast to plots with vegetation reaching higher height strata, which promoted small‐bodied communities with largely insectivorous diets (negative PC1).

### 3.2 Avian Diversity‐Vegetation Structure Associations

**FIGURE 5:**
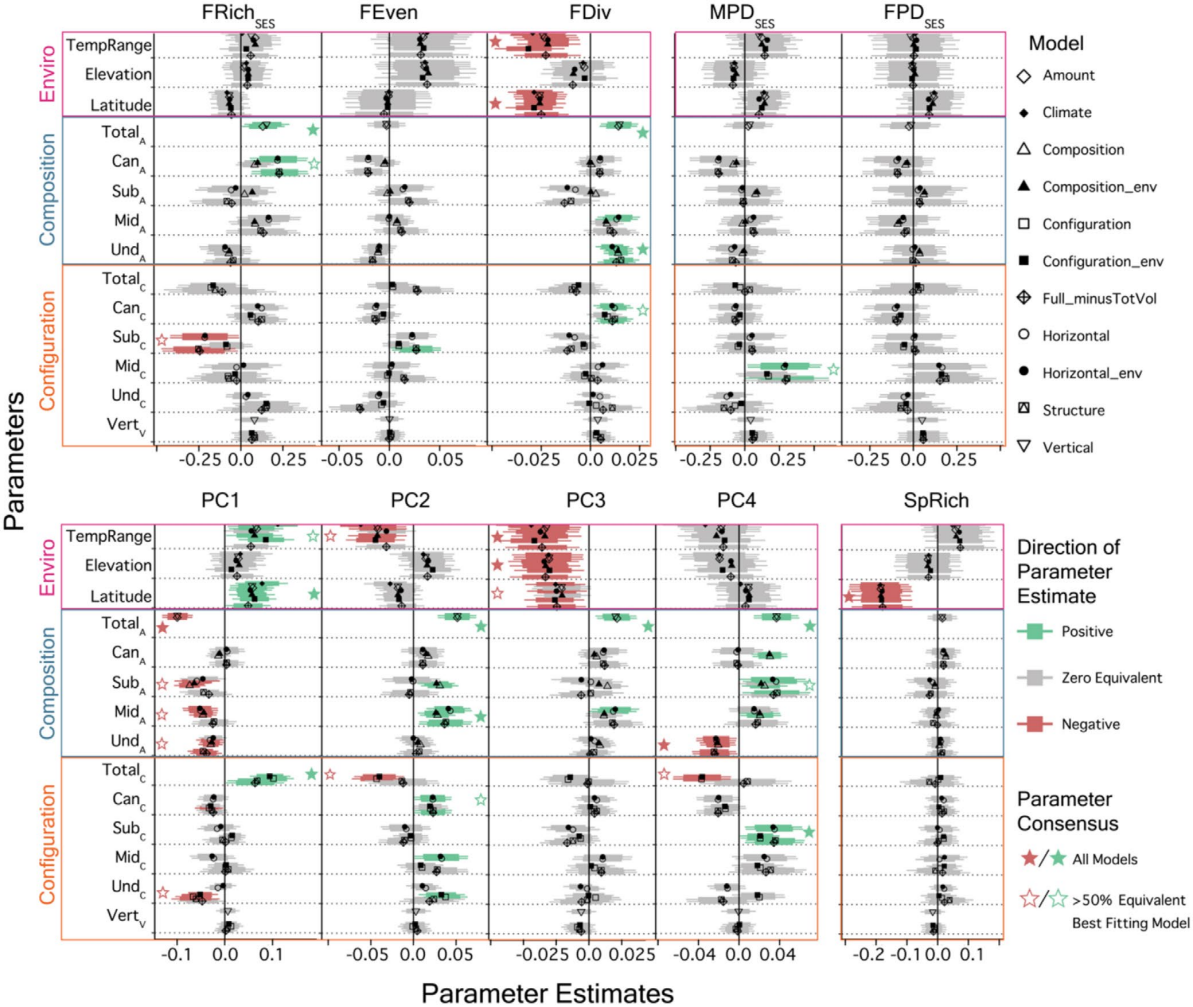
Parameter estimates from each model (excluding full model) for all 10 biodiversity response variables (SpRich = species richness, PD=Faith's Phylogenetic Diversity, MPD = mean pairwise distance, FRich = Functional Richness, FEven = Functional Evenness, FDiv = Functional Divergence, PC1 = Principal Coordinate 1, PC2 = Principal Coordinate 2, PC3 = Principal Coordinate 3, PC4 = Principal Coordinate 4). Boxes and tails around parameter estimates designate 80% and 95% credible intervals (CIs), respectively. Positive and negative parameter estimates whose 95% CIs do not overlap zero are shown in green and red, respectively. Parameter estimates whose 95% CIs overlap zero are shown in gray. Colored outlines designate parameters in broad categories of environmental (pink), habitat composition (blue), and habitat configuration (orange). Solid stars were added to parameter estimates when all models estimated either all positive or all negative estimates above a 95% CI for a particular response variable. Open stars were added when the best fitting model and at least 50% of models statistically equivalent to the best fitting model estimated positive or negative parameter values above a 95% CI.

3D habitat structure overall had no or a minor effect on species richness and phylogenetic diversity, with only MPD_SES_ showing a positive association with Mid_C_. In contrast, functional diversity showed strong associations with 3D habitat structure. Specifically, FRich_SES_ increased with Total_A_ and Can_A_ and decreased with Sub_C_ (Figure 5), indicative of strong increases in the breadth of the functional space of avian communities with increasing habitat amount and vegetation homogenization at higher vegetation strata. FDiv increased with Total_A_, Mid_A_, and Und_A_, and with Can_C_, indicating that increased habitat amount at lower vegetation strata but more patchy canopies lead to more functionally dispersed communities. However, FEven only increased with Sub_C_, suggesting that patchy vegetation at the subcanopy level leads to communities where species' abundances are more regularly distributed within the functional space (Figure 5).

PC trait axes showed strong associations with 3D habitat structure. In terms of 3D habitat composition, PC1 decreased with Total_A_, Sub_A_, Mid_A_, and Und_A_ (Figure 5), suggesting that high 3D habitat amount leads to assemblages with on average smaller body mass and beak size and an increased prevalence of traits such as insectivorous diet and mid‐height foraging, but decreased prevalence of traits associated with aquatic foraging and piscivorous diets. PC2 increased with Total_A_, Sub_A_, and Mid_A_ (Figure 5), suggesting that greater 3D habitat amount supports on average larger HWI and increased prevalence of aerial and below water foraging, but leads to decreased prevalence of ground foraging and granivorous diets. PC3 increased with Total_A_ and Mid_A_ (Figure 5), suggesting that more 3D habitat amount leads to increased prevalence of herbivore diets and decreased prevalence of nectarivore diets. Total_A_, Can_A_, Sub_A_, and Mid_A_ were all positively related to PC4, suggesting that more 3D habitat amount leads to increased prevalence of canopy foraging, frugivore diets, scavenger diets, endotherm diets, ectotherm diets, wider and taller beaks, and decreased prevalence of understory foraging. However, PC4 decreased with Und_A_, indicating that understory amount supported the opposite traits than Can_A_, Sub_A_, and Mid_A_ (Figure 5).

In terms of habitat configuration, Total_C_ (i.e., 2D habitat configuration) and strata‐specific configuration indices showed opposite relationships with PC axes. PC1 increased with Total_C_ (Figure 5), suggesting that increased 2D habitat heterogeneity leads to assemblages with on average larger body mass and beak dimensions, increases the prevalence of water surface foraging and piscivorous diets, but decreases the prevalence of insectivore diets and mid‐height foraging. In contrast, PC1 decreased with Can_C_ and Und_C_. PC2 decreased with Total_C_ (Figure 5), indicating that 2D habitat heterogeneity leads to increased prevalence of ground foraging and granivore diets and decreased prevalence of aerial and below‐water foraging. PC2 decreased with Und_C_, Mid_C_, and Can_C_. PC3 showed no relationships with either 2D or 3D habitat configuration. PC4 decreased with Total_C_ (Figure 5), indicating that 2D habitat heterogeneity leads to increased prevalence of understory foraging but decreased prevalence of canopy foraging, frugivore, scavenger, endotherm, and ectotherm diets, as well as narrower and shallower beaks. In contrast, PC4 increased with Sub_C_ (Figure 5).

## 4 Discussion

Our study is one of the first to evaluate the associations of a comprehensive suite of 3D habitat composition and configuration metrics with, not only measures of avian species richness, but also phylogenetic, trait, and functional diversity, and to contrast the effects of 3D habitat configuration with those of 2D habitat configuration. Our findings illustrate that although total 3D habitat amount emerged as the most consistent predictor of avian functional and trait‐based diversity, it did not have a strong effect on species richness and phylogenetic diversity, thus offering only partial support to our first hypothesis. Functional and trait‐based diversity, however, showed the strongest relationships with 3D habitat structure, supporting our second hypothesis.

### 4.1 Effects of 3D Habitat Composition on Avian Diversity

Increases in 3D habitat amount correspond to an expansion of functional space, which is consistent with studies that have demonstrated a strong relationship between canopy height and functional space (MacArthur and MacArthur 1961; Feng et al. 2020; Remeš et al. 2021). This expansion of functional space along the gradient of 3D habitat amount is likely driven by the addition of more species that exhibit extreme trait values that are positioned farther away from the functional space centroid, as suggested by the positive relationship between habitat amount and avian functional divergence. Interestingly, despite the expansion of functional space with increasing habitat amount, functional evenness remains stable, suggesting that species and abundances are added to functional space in a somewhat regular manner as vegetation amount increases.

Increased vegetation amount led to communities that, on average, foraged at higher vertical levels and had broader spectrum of dietary characteristics, which supports the notion that increases in 3D habitat amount lead to increases in available niche space. On the other hand, communities in locations with lower habitat amount were composed of more ground foraging species that consumed a higher proportion of seeds and nectar in their diet. Intuitively, this result aligns well with the notion that grasslands and other types of open habitats support greater proportions of seed and flower‐producing flora such as grasses and forbs. In partial contrast, increasing amount of understory habitat did not lead to higher foraging communities but instead was associated with increased understory foraging but reduced canopy foraging. However, this result is in line with previous findings that show that, while understory vegetation provides cover to low foraging birds (Tallei et al. 2021), closed canopies suppress the growth of understory plants (Svenning 2000; van Pelt and Franklin 2000), indicating that increased canopy amount limits species foraging in understory.

Increases in 3D habitat composition were also associated with communities composed of, on average, smaller species and those with higher HWI. This observation makes sense given that smaller species often require denser vegetative cover to hide from predators (Stirnemann et al. 2015) and are perhaps able to maneuver more easily in dense vegetation than larger birds (Provini and Höfling 2020). HWI is closely correlated with migration ability (Sheard et al. 2020), suggesting that increases in 3D habitat amount promote communities comprised of higher proportions of, on average, migratory birds. This trend aligns with findings that migratory birds tend to be smaller (Soriano‐Redondo et al. 2020), have a larger portion of their diet comprised of seasonally available foods such as fruit and insects, and are more likely to forage in the canopy compared to resident bird species (Levey and Stiles 1992; Boyle et al. 2011). Through the creation of more seasonally available foods and suitable habitats, increased 3D habitat amount might thus attract species characterized by increased migration ability and smaller body size.

### 4.2 Effects of 2D and 3D Habitat Configuration on Avian Diversity

Increased heterogeneity (patchiness) of the subcanopy level decreases the breadth of the functional space but increases the regularity of species abundances in that space. More patchy canopies were also shown to support more functionally divergent communities than homogenous canopies, indicating that species with more extreme trait values are often highly abundant in patchy canopies. While these findings for subcanopy and canopy configuration may initially seem contradictory, they make sense if: as upper vertical strata become patchier, low‐abundant species occupying the periphery of the functional space disappear from the assemblage, leaving abundant and functionally unique species more evenly distributed within the remaining trait space. Abiotic edge effects found in patchy habitats, such as more extreme microclimates (Fischer and Lindenmayer 2007), likely plays a role in the removal of low‐abundant peripheral species via environmental filtering. However, the absence of changes in functional space across lower height strata suggests that higher vertical strata have different types of environmental filtering compared to lower height strata.

Higher 3D habitat heterogeneity was associated with foraging in higher strata, higher proportions of species with carnivorous diets, and larger HWI. Increased openness of the vegetation profile, especially at higher height strata, thus appears to facilitate predatory species and increased flying ability. Previous research indeed suggests that open spaces at high strata support raptors (Swolgaard et al. 2008), perhaps by providing greater foraging opportunities facilitated by increased maneuverability or improved habitat for prey species. This observation is further supported by research on 2D habitat configuration that shows greater predation rates from raptors and other predators in more heterogenous habitats compared to more homogenous ones (Preston 1990; Batary and Baldi 2004). Moreover, species with larger HWI tend to have a greater gap‐crossing ability (Claramunt et al. 2022), perhaps allowing them to easily maneuver and persist in patchy habitats (but see Jones et al. 2023). Indeed, larger HWI has been found to be more prevalent in areas with increased disturbance (Claramunt et al. 2022; Naka et al. 2022; Weeks et al. 2023). An alternative explanation for the increase in assemblage‐level HWI in areas with increased 3D habitat configuration could be an increase in the prevalence of migratory species with insectivorous diets, which are known to increase along forest edges (Terraube et al. 2016). Forest edges tend to support higher abundances of herbivorous insects (Barbosa et al. 2005), meaning that migratory insectivorous bird species—which have larger HWI in temperate regions (Sheard et al. 2020)—might simply be attracted to areas with higher prey availability.

Interestingly, 2D habitat configuration had largely the opposite effect on individual trait characteristics compared to 3D habitat configuration. Increases in 2D habitat heterogeneity led to assemblages comprised of proportionally more ground and understory foraging species, higher prevalence of granivorous diets, aquatic‐associated traits, and on average larger body mass. Granivores and ground foraging species have been shown to be less sensitive to 2D habitat heterogeneity, with changes in resource availability between disturbed and non‐disturbed habitats being a key driver of the observed differences in trait responses (Kennedy et al. 2010). Therefore, species that forage at higher strata or belong to non‐granivorous dietary guilds may be more sensitive to disturbance and thus less represented in areas of high 2D habitat heterogeneity.

The differing effects of 2D and 3D habitat configuration on bird assemblages might stem from the fact that 3D configuration captures gaps in vegetation within a particular height stratum, while vegetation may still be present above or below. In contrast, 2D configuration only captures gaps when vegetation is absent across all vertical strata. Metrics of 2D configuration are thus much more likely to capture hard edges, an abrupt boundary between habitat types (Malt and Lank 2007), which often includes open habitats, such as grasslands, that favor lower foraging and granivorous diets (Wiens and Rotenberry 1979). We suggest that measures of 2D configuration lack the ability to capture internal structural heterogeneity as effectively as measures of 3D habitat configuration. While 3D configuration more accurately measures the effects of habitat arrangement and the effects of open space at different height strata, 2D configuration inherently captures aspects of habitat mosaicism.

In contrast to previous studies (MacArthur and MacArthur 1961; Huang et al. 2014; Carrasco et al. 2019; Moudrý et al. 2021, 2023), we did not find any associations between avian diversity and habitat configuration along the entire vertical profile. Our chosen metric, variance of the number of voxels across the vegetation profile, was largely independent of 3D habitat composition and demonstrated no discernible relationships with avian diversity. Our findings suggest that configuration of the entire vertical profile may be less relevant for birds when measured independent of habitat amount, at least at the level of entire communities. This may be because most bird movement is not strictly vertical, instead occurring both horizontally and vertically. Another potential explanation is that variance, like many metrics of variation, might take the same value under two different scenarios: it would be low in areas with very little vegetation and in areas with full foliage across the entire profile. Indeed, the highest levels of variance occurred at plots from predominantly grassland or scrubland regions where the presence of some amount of sparse vegetation appears to yield much higher values of variance compared to forested sites (see Figure S3).

### 4.3 On the Lack of Relationship With Species Richness

Interestingly, we found little to no associations between 3D vegetation structure and species richness or phylogenetic diversity. Initially, we were puzzled by this lack of relationship, especially given the known links between richness and vegetation structure (Lesak et al. 2011; Feng et al. 2020; Burns et al. 2020; Xu et al. 2024). However, we believe this result may not be entirely inconsistent with current knowledge. Our analyses included sites spanning various habitat types, from grasslands to forests. Consequently, our metrics of 3D habitat amount are likely to be higher in forested regions compared to areas with less vegetation. Yet, there is no consistent relationship between habitat type and bird diversity (Basile et al. 2021). For instance, Jarzyna et al. (2015) found that species richness was, on average, lower in the forested regions of New York than in agricultural mosaics. By focusing on multiple habitat types, we may not have been able to disentangle the specific effects of vegetation structure on species richness. We recommend that future studies focus on specific habitat types and their unique structural characteristics to better understand their individual effects on species richness and phylogenetic diversity.

## 5 Conclusions

Among various facets of avian diversity, functional and trait diversity are most reliably influenced by 3D habitat structure, implying that the relationship between avian diversity and vegetation structure is mediated through species traits, at least at a local scale equivalent to NEON survey plots. Total 3D habitat amount emerged as the most reliable predictor of functional and trait‐based diversity, suggesting that 3D habitat composition plays an outsized role in shaping avian community diversity, likely through an increase in niche space. Importantly, both habitat composition and habitat configuration had negative, positive, and neutral effects on avian diversity across different height strata and between 2D and 3D measures of habitat configuration. These mixed findings reveal that: (1) different processes can operate along different height strata, and (2) the findings of studies on habitat structure are sensitive to how structural indices are defined. By carefully considering relationships between multi‐faceted avian diversity and both compositional and configurational 3D habitat structure, our work provides a framework for addressing the inconsistencies that have historically troubled studies on the interplay between biodiversity and vegetation structure.

## Author Contributions

**Colin P. Sweeney:** conceptualization (equal), data curation (lead), formal analysis (lead), investigation (lead), methodology (equal), software (lead), visualization (lead), writing – original draft (lead), writing – review and editing (lead). **William Peterman:** conceptualization (supporting), methodology (supporting), software (supporting), writing – review and editing (supporting). **Kaiguang Zhao:** conceptualization (supporting), methodology (supporting), software (supporting), writing – review and editing (supporting). **Karen Goodell:** conceptualization (supporting), methodology (supporting), writing – review and editing (supporting). **Benjamin Zuckerberg:** writing – review and editing (supporting). **Marta A. Jarzyna:** conceptualization (equal), data curation (supporting), formal analysis (supporting), investigation (supporting), methodology (equal), project administration (lead), resources (lead), software (supporting), supervision (lead), validation (supporting), writing – original draft (supporting), writing – review and editing (supporting).

## Conflicts of Interest

The authors declare no conflicts of interest.

## Supporting information


Appendix S1:

## Data Availability

All derived data used to create this publication have been made publicly available on https://doi.org/10.5061/dryad.t76hdr87q with links to all other public data repositories already in use. Annotated code used in these analyses is also available on GitHub at the following link https://github.com/ColinPSweeney/3D‐habitat‐structure‐drives‐avian‐functional‐and‐trait‐diversity‐across‐North‐America.git.
