# Supplementary material for: Three‐Dimensional Habitat Structure Drives Avian Functional and Trait Diversity Across North America
Source: Ecol Evol. 2025 Apr 23;15(4):e70988. doi: 10.1002/ece3.70988 (PMC12015643; doi:10.1002/ece3.70988)
Supplement: Supplementary file 1 — Appendix S1: [file ECE3-15-e70988-s001.docx]

**Title**: 3D habitat structure drives avian functional and trait diversity across North America

**Supporting Information**

Tables and Figures:


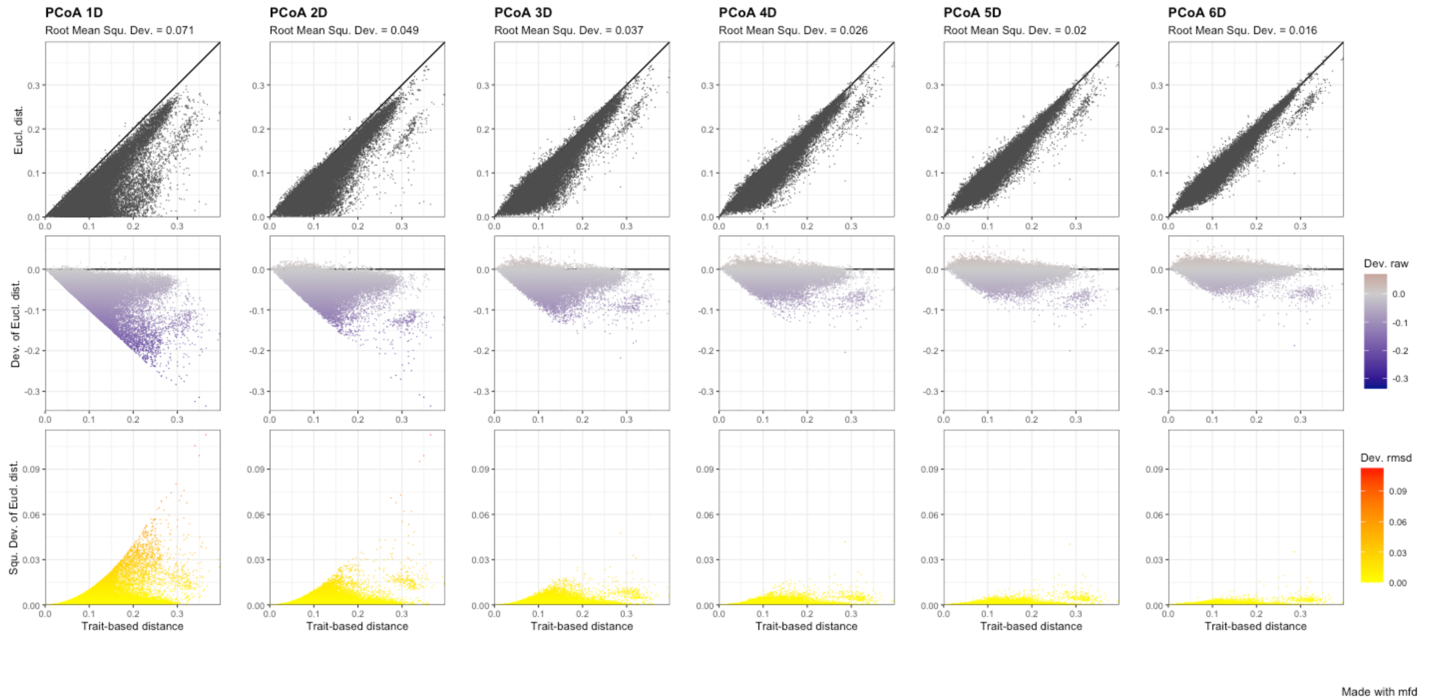


***Fig. S1 |*** Diagnostic plots for PC axes, labeled here as PCoA axes, generated from the “mFD” package using the “quality.fspaces.plot” function. For Euclidian distance (Eucl. dist.), deviation of Euclidian distance (Dev. of Eucl. dist.) and squared deviation of scaled Euclidian distance (Squ. Dev. of scaled Eucl. Dist.), a noticeable improvement in fit occurred at the PC4, with less improvement of fit and deviation at the two higher axes shown (PC5 and PC6). Diminishing improvement past PC4 allowed for the selection of 4 PC axes for data analysis.


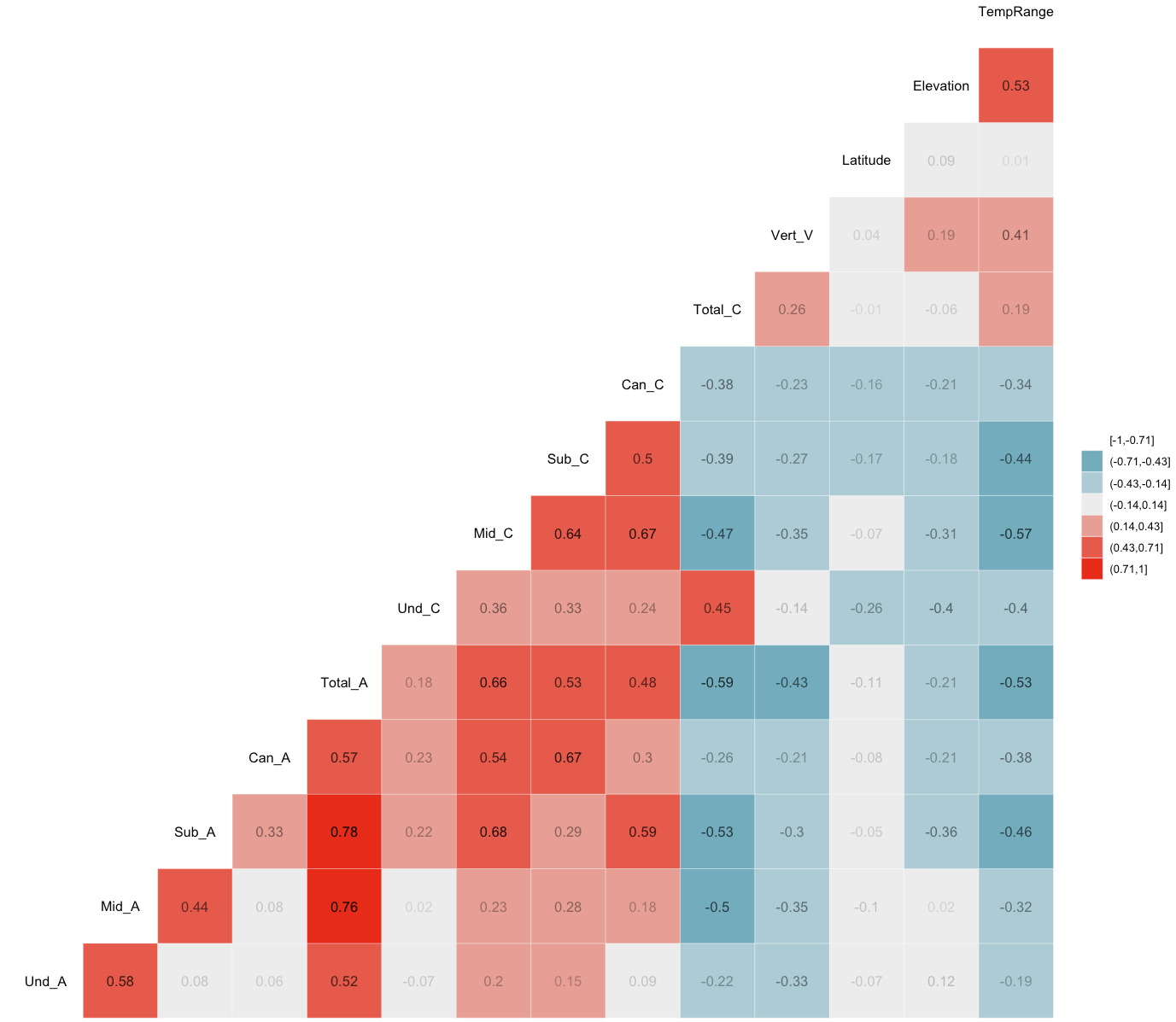


***Fig. S2 |*** Pearson's product-moment correlation coefficient *r* among the variable include in our study. Correlations of habitat structure indices were calculated using data from all avian plots used in the study. The suffix “C” indicates configuration and the suffix “A” indicates (composition). “Und”, “Mid”, “Sub”, and “Can” indicate understory, midstory, subcanopy, and canopy strata, respectively, while “Total” represents all strata combined. “Vert_V” represents vertical variance. A correlation greater than 0.7 was considered as indicative of high correlation.


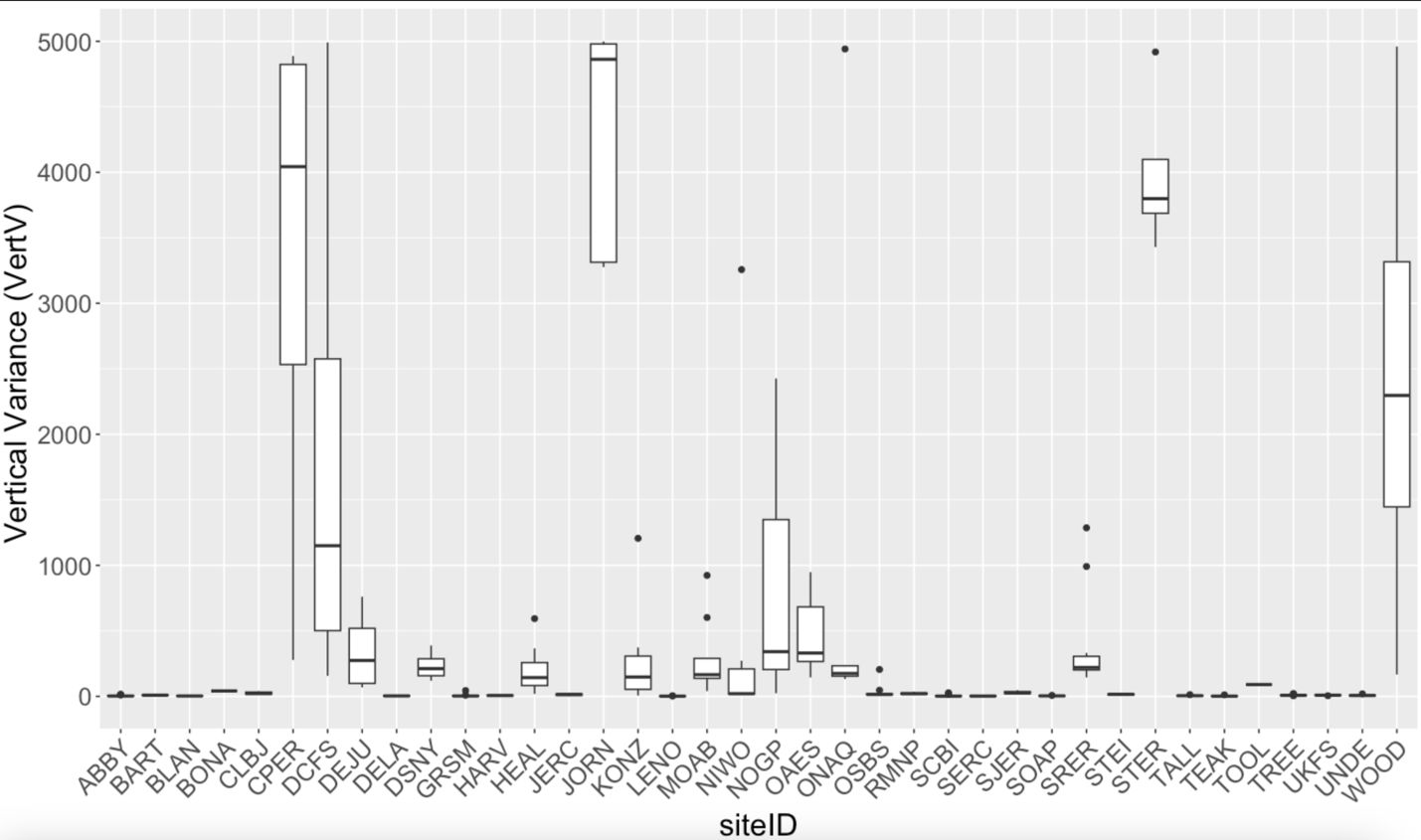


***Fig. S3 |*** Figure shows boxplots of each NEON site and the Vertical variance (Vert_V_) t of avian plots. Sites without large changes in Vertical variance amount between avian plots appear as lines.

***Table S1 |*** Table of avian survey dates for each of the NEON sites for 2017.

| domainID | siteID | StartDate | EndDate | numPlots |
| --- | --- | --- | --- | --- |
| D01 | BART | 2017-06-21 | 2017-06-29 | 9 |
| D01 | HARV | 2017-06-02 | 2017-06-15 | 12 |
| D02 | BLAN | 2017-05-24 | 2017-06-06 | 7 |
| D02 | SCBI | 2017-05-15 | 2017-06-09 | 17 |
| D02 | SERC | 2017-05-15 | 2017-06-05 | 17 |
| D03 | DSNY | 2017-05-15 | 2017-05-20 | 11 |
| D03 | JERC | 2017-05-23 | 2017-05-28 | 9 |
| D03 | OSBS | 2017-05-10 | 2017-05-14 | 10 |
| D05 | STEI | 2017-06-02 | 2017-06-13 | 10 |
| D05 | TREE | 2017-06-14 | 2017-06-27 | 11 |
| D05 | UNDE | 2017-06-09 | 2017-06-21 | 11 |
| D06 | KONZ | 2017-06-03 | 2017-06-10 | 12 |
| D06 | UKFS | 2017-06-11 | 2017-06-15 | 5 |
| D07 | GRSM | 2017-05-25 | 2017-06-04 | 15 |
| D08 | DELA | 2017-06-23 | 2017-06-27 | 9 |
| D08 | LENO | 2017-06-19 | 2017-06-21 | 15 |
| D08 | TALL | 2017-06-09 | 2017-06-17 | 15 |
| D09 | DCFS | 2017-06-27 | 2017-07-07 | 13 |
| D09 | NOGP | 2017-07-08 | 2017-07-11 | 13 |
| D09 | WOOD | 2017-07-06 | 2017-07-12 | 9 |
| D10 | CPER | 2017-05-26 | 2017-06-05 | 15 |
| D10 | RMNP | 2017-06-26 | 2017-07-08 | 12 |
| D10 | STER | 2017-05-17 | 2017-05-24 | 6 |
| D11 | CLBJ | 2017-05-09 | 2017-05-22 | 8 |
| D11 | OAES | 2017-05-26 | 2017-06-01 | 7 |
| D13 | MOAB | 2017-05-23 | 2017-05-29 | 10 |
| D13 | NIWO | 2017-07-11 | 2017-07-17 | 6 |
| D14 | JORN | 2017-04-27 | 2017-05-06 | 10 |
| D14 | SRER | 2017-05-03 | 2017-05-13 | 12 |
| D15 | ONAQ | 2017-05-19 | 2017-05-22 | 6 |
| D16 | ABBY | 2017-05-26 | 2017-06-05 | 20 |
| D17 | SJER | 2017-04-12 | 2017-04-25 | 10 |
| D17 | SOAP | 2017-05-16 | 2017-05-19 | 13 |
| D17 | TEAK | 2017-06-16 | 2017-06-30 | 12 |
| D18 | TOOL | 2017-06-21 | 2017-06-26 | 1 |
| D19 | BONA | 2017-06-13 | 2017-06-23 | 1 |
| D19 | DEJU | 2017-06-13 | 2017-06-18 | 4 |
| D19 | HEAL | 2017-06-05 | 2017-06-12 | 7 |

***Table S2 |*** Loo compare outputs for each set of 12 Bayesian Hierarchical Distance Sampling (BHDS) models organized by biodiversity response variable. Ranking indicates which model performed best according to Loo compare output. Model name indicates which of the 12 models (see table 1 in main text for formulation). Model Icon represents the legend icons from Figure 4 in the main text (included to enhance interpretability and cross referencing). Full models were not included in Figure 4 and thus did not receive an icon here. Elpd_diff is the difference in expected log pointwise predictive density between the top performing model and the comparison model. Se_diff is the standard error of component-wise differences of elpd_loo between the top performing model and the model of comparison. Diff Range (+) and Diff Range (-) are either the elpd_diff with se_diff added or subtracted respectively. The column labeled “3 Times rule” indicates if the absolute value of elpd_diff is at least three times larger than se_diff. If abs(elpd_diff) is three times larger, the comparison model is considered to be not equivalent in fit to the top performing model and is labeled “Not Equivalent”. If abs(elpd_diff) is not three times larger, then the comparison model is considered to have roughly the same level of fit as the top performing model and both are used equally to inform interpretation of results. Models considered to have equal fit to the top performing model are labeled as “Equivalent”. In practice, LOO comparisons found no significant difference between top performing models and those with parameter estimates of >95% credible intervals.

| \| SpRich \| \|  \|  \|  \|  \|  \|  \|  \| \| --- \| --- \| --- \| --- \| --- \| --- \| --- \| --- \| --- \| \| **Ranking** \| \| **Model name** \| **Model Icon** \| **elpd_diff** \| **se_diff** \| **Diff Range** \| **Diff Range** \| **3 Times rule** \| \| 1 \| \| spRich _Climate \| 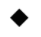 \| 0 \| 0 \| (+) \| (-) \|  \| \| 2 \| \| spRich _amount \| 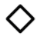 \| -0.9 \| 0.6 \| -0.3 \| -1.5 \| Equivalent \| \| 3 \| \| spRich _Vertical \| 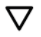 \| -1.4 \| 0.8 \| -0.6 \| -2.2 \| Equivalent \| \| 4 \| \| spRich _composition_env \| 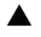 \| -2.9 \| 1.4 \| -1.5 \| -4.3 \| Equivalent \| \| 5 \| \| spRich_configuration_env \| 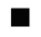 \| -3.2 \| 1.6 \| -1.6 \| -4.8 \| Equivalent \| \| 6 \| \| spRich _horizontal_env \| 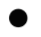 \| -5 \| 1.7 \| -3.3 \| -6.7 \| Equivalent \| \| 7 \| \| **spRich _full_minusTotVol** \| 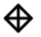 \| -6.3 \| 1.8 \| -4.5 \| -8.1 \| Not Equivalent \| \| 8 \| \| **spRich _full** \| **NA** \| -6.3 \| 1.8 \| -4.5 \| -8.1 \| Not Equivalent \| \| 9 \| \| spRich_composition \| 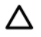 \| -6.5 \| 2.6 \| -3.9 \| -9.1 \| Equivalent \| \| 10 \| \| spRich _configuration \| 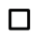 \| -7.1 \| 2.8 \| -4.3 \| -9.9 \| Equivalent \| \| 11 \| \| spRich _horizontal \| 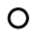 \| -8.5 \| 2.9 \| -5.6 \| -11.4 \| Equivalent \| \| 12 \| \| **spRich_structure** \| 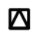 \| -9.7 \| 2.9 \| -6.8 \| -12.6 \| Not Equivalent \| \|  \| \|  \|  \|  \|  \|  \|  \|  \| \|  \| FRich_SES \| \| \| \| \| \| \| \| \| **Ranking** \| \| **Model name** \| **Model Icon** \| **elpd_diff** \| **se_diff** \| **Diff Range** \| **Diff Range** \| **3 Times rule** \| \| 1 \| \| FRic _amount \| 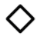 \| 0 \| 0 \| (+) \| (-) \|  \| \| 2 \| \| FRic _Vertical \| 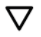 \| -0.2 \| 1.3 \| 1.1 \| -1.5 \| Equivalent \| \| 3 \| \| FRic _composition \| 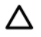 \| -0.8 \| 2.6 \| 1.8 \| -3.4 \| Equivalent \| \| 4 \| \| FRic _Climate \| 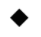 \| -1 \| 2.1 \| 1.1 \| -3.1 \| Equivalent \| \| 5 \| \| FRic_composition_env \| 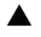 \| -1.5 \| 1.7 \| 0.2 \| -3.2 \| Equivalent \| \| 6 \| \| FRic _horizontal \| 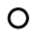 \| -2.1 \| 3.4 \| 1.3 \| -5.5 \| Equivalent \| \| 7 \| \| FRic _configuration \| 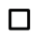 \| -2.6 \| 2.8 \| 0.2 \| -5.4 \| Equivalent \| \| 8 \| \| FRic _structure \| 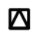 \| -2.9 \| 3.5 \| 0.6 \| -6.4 \| Equivalent \| \| 9 \| \| FRic _horizontal_env \| 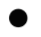 \| -3.2 \| 2.8 \| -0.4 \| -6 \| Equivalent \| \| 10 \| \| FRic _configuration_env \| 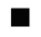 \| -4.3 \| 2.3 \| -2 \| -6.6 \| Equivalent \| \| 11 \| \| FRic_full_minusTotVol \| 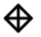 \| -4.3 \| 3 \| -1.3 \| -7.3 \| Equivalent \| \| 12 \| \| FRic _full \| **NA** \| -4.3 \| 3 \| -1.3 \| -7.3 \| Equivalent \| \|  \| \|  \|  \|  \|  \|  \|  \|  \| \| FEven \| \|  \|  \|  \|  \|  \|  \|  \| \| **Ranking** \| \| **Model name** \| **Model Icon** \| **elpd_diff** \| **se_diff** \| **Diff Range** \| **Diff Range** \| **3 Times rule** \| \| 1 \| \| FEve _Climate \| 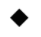 \| 0 \| 0 \| (+) \| (-) \|  \| \| 2 \| \| **FEve _amount** \| 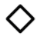 \| -1.8 \| 0.4 \| -1.4 \| -2.2 \| Not Equivalent \| \| 3 \| \| **FEve _Vertical** \| 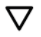 \| -2.5 \| 0.4 \| -2.1 \| -2.9 \| Not Equivalent \| \| 4 \| \| FEve_composition_env \| 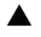 \| -2.8 \| 1.8 \| -1 \| -4.6 \| Equivalent \| \| 5 \| \| FEve _composition \| 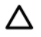 \| -3 \| 1.8 \| -1.2 \| -4.8 \| Equivalent \| \| 6 \| \| FEve _horizontal \| 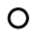 \| -5 \| 3.7 \| -1.3 \| -8.7 \| Equivalent \| \| 7 \| \| FEve _horizontal_env \| 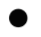 \| -5.8 \| 3.7 \| -2.1 \| -9.5 \| Equivalent \| \| 8 \| \| FEve _full_minusTotVol \| 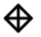 \| -5.9 \| 3.9 \| -2 \| -9.8 \| Equivalent \| \| 9 \| \| FEve _structure \| 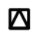 \| -6.4 \| 3.9 \| -2.5 \| -10.3 \| Equivalent \| \| 10 \| \| **FEve _configuration** \| 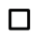 \| -6.5 \| 2 \| -4.5 \| -8.5 \| Not Equivalent \| \| 11 \| \| FEve _full \| **NA** \| -6.5 \| 3.9 \| -2.6 \| -10.4 \| Equivalent \| \| 12 \| \| FEve _configuration_env \| 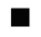 \| -7.1 \| 1.8 \| -5.3 \| -8.9 \| Not Equivalent \| \|  \| \|  \|  \|  \|  \|  \|  \|  \| \| Fdiv \| \|  \|  \|  \|  \|  \|  \|  \| \| **Ranking** \| \| **Model name** \| **Model Icon** \| **elpd_diff** \| **se_diff** \| **Diff Range** \| **Diff Range** \| **3 Times rule** \| \| 1 \| \| FDiv _composition \| 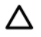 \| 0 \| 0 \| (+) \| (-) \|  \| \| 2 \| \| FDiv _structure \| 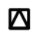 \| -0.3 \| 3 \| 2.7 \| -3.3 \| Equivalent \| \| 3 \| \| FDiv _composition_env \| 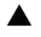 \| -0.6 \| 1.8 \| 1.2 \| -2.4 \| Equivalent \| \| 4 \| \| FDiv_full_minusTotVol \| 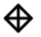 \| -0.9 \| 3.5 \| 2.6 \| -4.4 \| Equivalent \| \| 5 \| \| FDiv _horizontal_env \| 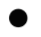 \| -1 \| 3 \| 2 \| -4 \| Equivalent \| \| 6 \| \| FDiv _horizontal \| 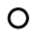 \| -1.1 \| 2.7 \| 1.6 \| -3.8 \| Equivalent \| \| 7 \| \| FDiv _full \| **NA** \| -1.2 \| 3.5 \| 2.3 \| -4.7 \| Equivalent \| \| 8 \| \| FDiv _Vertical \| 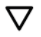 \| -5.2 \| 5 \| -0.2 \| -10.2 \| Equivalent \| \| 9 \| \| FDiv _amount \| 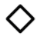 \| -5.4 \| 4.6 \| -0.8 \| -10 \| Equivalent \| \| 10 \| \| FDiv _Climate \| 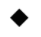 \| -7.3 \| 6.1 \| -1.2 \| -13.4 \| Equivalent \| \| 11 \| \| FDiv_configuration \| 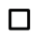 \| -9.5 \| 6.8 \| -2.7 \| -16.3 \| Equivalent \| \| 12 \| \| FDiv _configuration_env \| 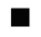 \| -10 \| 6.7 \| -3.3 \| -16.7 \| Equivalent \| \|  \| \|  \|  \|  \|  \|  \|  \|  \| \| PC1 \| \|  \|  \|  \|  \|  \|  \|  \| \| **Ranking** \| \| **Model name** \| **Model Icon** \| **elpd_diff** \| **se_diff** \| **Diff Range** \| **Diff Range** \| **3 Times rule** \| \| 1 \| \| PC1 _amount \| 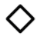 \| 0 \| 0 \| (+) \| (-) \|  \| \| 2 \| \| PC1 _composition_env \| 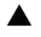 \| -0.3 \| 2 \| 1.7 \| -2.3 \| Equivalent \| \| 3 \| \| PC1 _Vertical \| 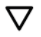 \| -0.5 \| 0.4 \| -0.1 \| -0.9 \| Equivalent \| \| 4 \| \| PC1 _composition \| 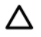 \| -1.3 \| 2.3 \| 1 \| -3.6 \| Equivalent \| \| 5 \| \| PC1_full_minusTotVol \| 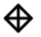 \| -2.6 \| 4.4 \| 1.8 \| -7 \| Equivalent \| \| 6 \| \| PC1_horizontal_env \| 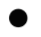 \| -2.9 \| 3.4 \| 0.5 \| -6.3 \| Equivalent \| \| 7 \| \| PC1 _full \| **NA** \| -2.9 \| 4.4 \| 1.5 \| -7.3 \| Equivalent \| \| 8 \| \| PC1 _structure \| 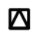 \| -3.2 \| 5.3 \| 2.1 \| -8.5 \| Equivalent \| \| 9 \| \| PC1 _horizontal \| 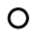 \| -3.3 \| 3.9 \| 0.6 \| -7.2 \| Equivalent \| \| 10 \| \| PC1 _configuration_env \| 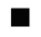 \| -7.4 \| 7.1 \| -0.3 \| -14.5 \| Equivalent \| \| 11 \| \| PC1 _configuration \| 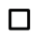 \| -9.5 \| 8.2 \| -1.3 \| -17.7 \| Equivalent \| \| 12 \| \| PC1 _Climate \| 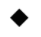 \| -12.8 \| 6.3 \| -6.5 \| -19.1 \| Equivalent \| \|  \| \|  \|  \|  \|  \|  \|  \|  \| \| PC2 \| \|  \|  \|  \|  \|  \|  \|  \| \| **Ranking** \| \| **Model name** \| **Model Icon** \| **elpd_diff** \| **se_diff** \| **Diff Range** \| **Diff Range** \| **3 Times rule** \| \| 1 \| \| PC2 _amount \| 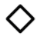 \| 0 \| 0 \| (+) \| (-) \|  \| \| 2 \| \| PC2 _Vertical \| 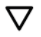 \| -3 \| 2.4 \| -0.6 \| -5.4 \| Equivalent \| \| 3 \| \| PC2 _full_minusTotVol \| 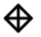 \| -3.2 \| 4 \| 0.8 \| -7.2 \| Equivalent \| \| 4 \| \| PC2 _horizontal \| 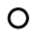 \| -3.3 \| 5 \| 1.7 \| -8.3 \| Equivalent \| \| 5 \| \| PC2 _full \| **NA** \| -3.6 \| 4 \| 0.4 \| -7.6 \| Equivalent \| \| 6 \| \| PC2_horizontal_env \| 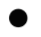 \| -3.9 \| 4.7 \| 0.8 \| -8.6 \| Equivalent \| \| 7 \| \| PC2 _composition \| 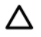 \| -4.4 \| 2.9 \| -1.5 \| -7.3 \| Equivalent \| \| 8 \| \| PC2 _composition_env \| 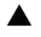 \| -4.9 \| 2.4 \| -2.5 \| -7.3 \| Equivalent \| \| 9 \| \| PC2 _structure \| 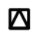 \| -5.3 \| 5.1 \| -0.2 \| -10.4 \| Equivalent \| \| 10 \| \| PC2 _Climate \| 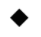 \| -5.7 \| 4.1 \| -1.6 \| -9.8 \| Equivalent \| \| 11 \| \| PC2 _configuration_env \| 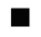 \| -5.8 \| 5.9 \| 0.1 \| -11.7 \| Equivalent \| \| 12 \| \| PC2_configuration \| 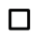 \| -6 \| 6.4 \| 0.4 \| -12.4 \| Equivalent \| \|  \| \|  \|  \|  \|  \|  \|  \|  \| \| PC3 \| \|  \|  \|  \|  \|  \|  \|  \| \| **Ranking** \| \| **Model name** \| **Model Icon** \| **elpd_diff** \| **se_diff** \| **Diff Range** \| **Diff Range** \| **3 Times rule** \| \| 1 \| \| PC3 _amount \| 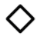 \| 0 \| 0 \| (+) \| (-) \|  \| \| 2 \| \| PC3_Vertical \| 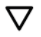 \| -0.5 \| 1 \| 0.5 \| -1.5 \| Equivalent \| \| 3 \| \| PC3 _composition \| 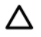 \| -2.3 \| 1.8 \| -0.5 \| -4.1 \| Equivalent \| \| 4 \| \| PC3 _Climate \| 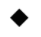 \| -2.3 \| 2.6 \| 0.3 \| -4.9 \| Equivalent \| \| 5 \| \| PC3 _composition_env \| 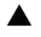 \| -2.4 \| 1.1 \| -1.3 \| -3.5 \| Equivalent \| \| 6 \| \| PC3 _horizontal \| 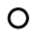 \| -7.2 \| 3 \| -4.2 \| -10.2 \| Equivalent \| \| 7 \| \| PC3 _horizontal_env \| 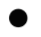 \| -7.3 \| 2.8 \| -4.5 \| -10.1 \| Equivalent \| \| 8 \| \| PC3 _configuration_env \| 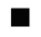 \| -8 \| 3.6 \| -4.4 \| -11.6 \| Equivalent \| \| 9 \| \| PC3 _configuration \| 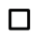 \| -8.8 \| 4.1 \| -4.7 \| -12.9 \| Equivalent \| \| 10 \| \| PC3 _full \| **NA** \| -9 \| 3 \| -6 \| -12 \| Equivalent \| \| 11 \| \| PC3 _full_minusTotVol \| 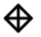 \| -9.2 \| 3.1 \| -6.1 \| -12.3 \| Equivalent \| \| 12 \| \| PC3 _structure \|  \| -9.3 \| 3.2 \| -6.1 \| -12.5 \| Equivalent \| \|  \| \|  \|  \|  \|  \|  \|  \|  \| \| PC4 \| \|  \|  \|  \|  \|  \|  \|  \| \| **Ranking** \| \| **Model name** \| **Model Icon** \| **elpd_diff** \| **se_diff** \| **Diff Range** \| **Diff Range** \| **3 Times rule** \| \| 1 \| \| PC4 _composition_env \| 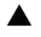 \| 0 \| 0 \| (+) \| (-) \|  \| \| 2 \| \| PC4_configuration \| 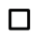 \| 0 \| 9.6 \| 9.6 \| -9.6 \| Equivalent \| \| 3 \| \| PC4 _configuration_env \| 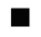 \| -0.4 \| 9 \| 8.6 \| -9.4 \| Equivalent \| \| 4 \| \| PC4 _composition \| 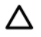 \| -0.6 \| 0.8 \| 0.2 \| -1.4 \| Equivalent \| \| 5 \| \| PC4 _horizontal_env \| 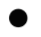 \| -2 \| 8 \| 6 \| -10 \| Equivalent \| \| 6 \| \| PC4 _horizontal \| 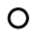 \| -2.2 \| 8.4 \| 6.2 \| -10.6 \| Equivalent \| \| 7 \| \| PC4 _structure \| 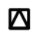 \| -4 \| 8.4 \| 4.4 \| -12.4 \| Equivalent \| \| 8 \| \| PC4_full_minusTotVol \| 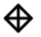 \| -4.3 \| 8 \| 3.7 \| -12.3 \| Equivalent \| \| 9 \| \| PC4 _full \| **NA** \| -4.3 \| 7.8 \| 3.5 \| -12.1 \| Equivalent \| \| 10 \| \| PC4 _amount \| 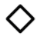 \| -4.4 \| 4.9 \| 0.5 \| -9.3 \| Equivalent \| \| 11 \| \| PC4 _Vertical \| 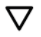 \| -5.2 \| 4.9 \| -0.3 \| -10.1 \| Equivalent \| \| 12 \| \| PC4 _Climate \| 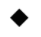 \| -5.6 \| 6.4 \| 0.8 \| -12 \| Equivalent \| \|  \| \|  \|  \|  \|  \|  \|  \|  \| \| FPD \| \|  \|  \|  \|  \|  \|  \|  \| \| **Ranking** \| \| **Model name** \| **Model Icon** \| **elpd_diff** \| **se_diff** \| **Diff Range** \| **Diff Range** \| **3 Times rule** \| \| 1 \| \| PD _Climate \| 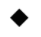 \| 0 \| 0 \| (+) \| (-) \|  \| \| 2 \| \| **PD_amount** \| 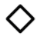 \| -1.2 \| 0.3 \| -0.9 \| -1.5 \| Not Equivalent \| \| 3 \| \| PD_Vertical \| 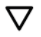 \| -1.5 \| 0.8 \| -0.7 \| -2.3 \| Equivalent \| \| 4 \| \| PD _configuration \| 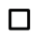 \| -1.9 \| 2.1 \| 0.2 \| -4 \| Equivalent \| \| 5 \| \| PD _composition \| 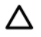 \| -2.2 \| 1.6 \| -0.6 \| -3.8 \| Equivalent \| \| 6 \| \| PD _composition_env \| 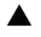 \| -2.8 \| 1.2 \| -1.6 \| -4 \| Equivalent \| \| 7 \| \| PD _configuration_env \| 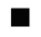 \| -3.4 \| 1.7 \| -1.7 \| -5.1 \| Equivalent \| \| 8 \| \| PD _horizontal \| 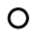 \| -4.2 \| 2.1 \| -2.1 \| -6.3 \| Equivalent \| \| 9 \| \| **PD _horizontal_env** \| 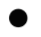 \| -5.7 \| 1.8 \| -3.9 \| -7.5 \| Not Equivalent \| \| 10 \| \| PD _structure \| 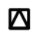 \| -5.8 \| 2.3 \| -3.5 \| -8.1 \| Equivalent \| \| 11 \| \| **PD _full_minusTotVol** \|  \| -7.5 \| 2 \| -5.5 \| -9.5 \| Not Equivalent \| \| 12 \| \| **PD _full** \| **NA** \| -7.5 \| 1.9 \| -5.6 \| -9.4 \| Not Equivalent \| \|  \| \|  \|  \|  \|  \|  \|  \|  \| \| MPD \| \|  \|  \|  \|  \|  \|  \|  \| \| **Ranking** \| \| **Model name** \| **Model Icon** \| **elpd_diff** \| **se_diff** \| **Diff Range** \| **Diff Range** \| **3 Times rule** \| \| 1 \| \| MPD _Climate \|  \| 0 \| 0 \| (+) \| (-) \|  \| \| 2 \| \| MPD _amount \|  \| -0.7 \| 0.3 \| -0.4 \| -1 \| Equivalent \| \| 3 \| \| MPD _Vertical \|  \| -1.6 \| 0.6 \| -1 \| -2.2 \| Equivalent \| \| 4 \| \| MPD_composition \|  \| -2.2 \| 1.7 \| -0.5 \| -3.9 \| Equivalent \| \| 5 \| \| MPD _configuration \|  \| -2.4 \| 2.5 \| 0.1 \| -4.9 \| Equivalent \| \| 6 \| \| MPD _horizontal \|  \| -2.7 \| 3.2 \| 0.5 \| -5.9 \| Equivalent \| \| 7 \| \| MPD _composition_env \|  \| -3 \| 1.1 \| -1.9 \| -4.1 \| Equivalent \| \| 8 \| \| MPD _configuration_env \|  \| -3.9 \| 2.1 \| -1.8 \| -6 \| Equivalent \| \| 9 \| \| MPD_horizontal_env \|  \| -4.5 \| 3 \| -1.5 \| -7.5 \| Equivalent \| \| 10 \| \| MPD _structure \|  \| -4.6 \| 3.3 \| -1.3 \| -7.9 \| Equivalent \| \| 11 \| \| MPD _full \| **NA** \| -5.6 \| 3.1 \| -2.5 \| -8.7 \| Equivalent \| \| 12 \| \| MPD _full_minusTotVol \|  \| -5.8 \| 3.1 \| -2.7 \| -8.9 \| Equivalent \| |
| --- | --- | --- | --- | --- | --- | --- | --- | --- | --- | --- | --- | --- | --- | --- | --- | --- | --- | --- | --- | --- | --- | --- | --- | --- | --- | --- | --- | --- | --- | --- | --- | --- | --- | --- | --- | --- | --- | --- | --- | --- | --- | --- | --- | --- | --- | --- | --- | --- | --- | --- | --- | --- | --- | --- | --- | --- | --- | --- | --- | --- | --- | --- | --- | --- | --- | --- | --- | --- | --- | --- | --- | --- | --- | --- | --- | --- | --- | --- | --- | --- | --- | --- | --- | --- | --- | --- | --- | --- | --- | --- | --- | --- | --- | --- | --- | --- | --- | --- | --- | --- | --- | --- | --- | --- | --- | --- | --- | --- | --- | --- | --- | --- | --- | --- | --- | --- | --- | --- | --- | --- | --- | --- | --- | --- | --- | --- | --- | --- | --- | --- | --- | --- | --- | --- | --- | --- | --- | --- | --- | --- | --- | --- | --- | --- | --- | --- | --- | --- | --- | --- | --- | --- | --- | --- | --- | --- | --- | --- | --- | --- | --- | --- | --- | --- | --- | --- | --- | --- | --- | --- | --- | --- | --- | --- | --- | --- | --- | --- | --- | --- | --- | --- | --- | --- | --- | --- | --- | --- | --- | --- | --- | --- | --- | --- | --- | --- | --- | --- | --- | --- | --- | --- | --- | --- | --- | --- | --- | --- | --- | --- | --- | --- | --- | --- | --- | --- | --- | --- | --- | --- | --- | --- | --- | --- | --- | --- | --- | --- | --- | --- | --- | --- | --- | --- | --- | --- | --- | --- | --- | --- | --- | --- | --- | --- | --- | --- | --- | --- | --- | --- | --- | --- | --- | --- | --- | --- | --- | --- | --- | --- | --- | --- | --- | --- | --- | --- | --- | --- | --- | --- | --- | --- | --- | --- | --- | --- | --- | --- | --- | --- | --- | --- | --- | --- | --- | --- | --- | --- | --- | --- | --- | --- | --- | --- | --- | --- | --- | --- | --- | --- | --- | --- | --- | --- | --- | --- | --- | --- | --- | --- | --- | --- | --- | --- | --- | --- | --- | --- | --- | --- | --- | --- | --- | --- | --- | --- | --- | --- | --- | --- | --- | --- | --- | --- | --- | --- | --- | --- | --- | --- | --- | --- | --- | --- | --- | --- | --- | --- | --- | --- | --- | --- | --- | --- | --- | --- | --- | --- | --- | --- | --- | --- | --- | --- | --- | --- | --- | --- | --- | --- | --- | --- | --- | --- | --- | --- | --- | --- | --- | --- | --- | --- | --- | --- | --- | --- | --- | --- | --- | --- | --- | --- | --- | --- | --- | --- | --- | --- | --- | --- | --- | --- | --- | --- | --- | --- | --- | --- | --- | --- | --- | --- | --- | --- | --- | --- | --- | --- | --- | --- | --- | --- | --- | --- | --- | --- | --- | --- | --- | --- | --- | --- | --- | --- | --- | --- | --- | --- | --- | --- | --- | --- | --- | --- | --- | --- | --- | --- | --- | --- | --- | --- | --- | --- | --- | --- | --- | --- | --- | --- | --- | --- | --- | --- | --- | --- | --- | --- | --- | --- | --- | --- | --- | --- | --- | --- | --- | --- | --- | --- | --- | --- | --- | --- | --- | --- | --- | --- | --- | --- | --- | --- | --- | --- | --- | --- | --- | --- | --- | --- | --- | --- | --- | --- | --- | --- | --- | --- | --- | --- | --- | --- | --- | --- | --- | --- | --- | --- | --- | --- | --- | --- | --- | --- | --- | --- | --- | --- | --- | --- | --- | --- | --- | --- | --- | --- | --- | --- | --- | --- | --- | --- | --- | --- | --- | --- | --- | --- | --- | --- | --- | --- | --- | --- | --- | --- | --- | --- | --- | --- | --- | --- | --- | --- | --- | --- | --- | --- | --- | --- | --- | --- | --- | --- | --- | --- | --- | --- | --- | --- | --- | --- | --- | --- | --- | --- | --- | --- | --- | --- | --- | --- | --- | --- | --- | --- | --- | --- | --- | --- | --- | --- | --- | --- | --- | --- | --- | --- | --- | --- | --- | --- | --- | --- | --- | --- | --- | --- | --- | --- | --- | --- | --- | --- | --- | --- | --- | --- | --- | --- | --- | --- | --- | --- | --- | --- | --- | --- | --- | --- | --- | --- | --- | --- | --- | --- | --- | --- | --- | --- | --- | --- | --- | --- | --- | --- | --- | --- | --- | --- | --- | --- | --- | --- | --- | --- | --- | --- | --- | --- | --- | --- | --- | --- | --- | --- | --- | --- | --- | --- | --- | --- | --- | --- | --- | --- | --- | --- | --- | --- | --- | --- | --- | --- | --- | --- | --- | --- | --- | --- | --- | --- | --- | --- | --- | --- | --- | --- | --- | --- | --- | --- | --- | --- | --- | --- | --- | --- | --- | --- | --- | --- | --- | --- | --- | --- | --- | --- | --- | --- | --- | --- | --- | --- | --- | --- | --- | --- | --- | --- | --- | --- | --- | --- | --- | --- | --- | --- | --- | --- | --- | --- | --- | --- | --- | --- | --- | --- | --- | --- | --- | --- | --- | --- | --- | --- | --- | --- | --- | --- | --- | --- | --- | --- | --- | --- | --- | --- | --- | --- | --- | --- | --- | --- | --- | --- | --- | --- | --- | --- | --- | --- | --- | --- | --- | --- | --- | --- | --- | --- | --- | --- | --- | --- | --- | --- | --- | --- | --- | --- | --- | --- | --- | --- | --- | --- | --- | --- | --- | --- | --- | --- | --- | --- | --- | --- | --- | --- | --- | --- | --- | --- | --- | --- | --- | --- | --- | --- | --- | --- | --- | --- | --- | --- | --- | --- | --- | --- | --- | --- | --- | --- | --- | --- | --- | --- | --- | --- | --- | --- | --- | --- | --- | --- | --- | --- | --- | --- | --- | --- | --- | --- | --- | --- | --- | --- | --- | --- | --- | --- | --- | --- | --- | --- | --- | --- | --- | --- | --- | --- | --- | --- | --- | --- | --- | --- | --- | --- | --- | --- | --- | --- | --- | --- | --- | --- | --- | --- | --- | --- | --- | --- | --- | --- | --- | --- | --- | --- | --- | --- | --- | --- | --- | --- | --- | --- | --- | --- | --- | --- | --- | --- | --- | --- | --- | --- | --- | --- | --- | --- | --- | --- | --- | --- | --- | --- | --- | --- | --- | --- | --- | --- | --- | --- | --- | --- | --- | --- | --- | --- | --- | --- | --- | --- | --- | --- | --- | --- | --- | --- | --- | --- | --- | --- | --- | --- | --- | --- | --- | --- | --- | --- | --- | --- | --- | --- | --- | --- | --- | --- | --- | --- | --- | --- | --- | --- | --- | --- | --- | --- | --- | --- | --- | --- | --- | --- | --- | --- | --- | --- | --- | --- | --- | --- | --- | --- | --- | --- | --- | --- | --- | --- | --- | --- | --- | --- | --- | --- | --- | --- | --- | --- | --- | --- | --- | --- | --- | --- | --- | --- | --- | --- | --- | --- | --- | --- | --- | --- | --- | --- | --- | --- | --- | --- | --- | --- | --- | --- | --- | --- | --- | --- | --- | --- | --- | --- | --- | --- | --- | --- | --- | --- | --- | --- | --- | --- | --- | --- | --- | --- | --- | --- | --- | --- | --- | --- | --- | --- | --- | --- | --- | --- | --- | --- | --- | --- | --- | --- | --- | --- | --- | --- | --- | --- | --- | --- | --- | --- | --- | --- | --- | --- | --- | --- | --- | --- | --- | --- | --- | --- | --- | --- | --- | --- | --- | --- | --- | --- | --- | --- | --- | --- | --- | --- | --- | --- | --- | --- | --- | --- | --- | --- | --- | --- | --- | --- | --- | --- | --- | --- | --- | --- | --- | --- | --- | --- | --- | --- | --- | --- | --- | --- | --- | --- | --- | --- | --- | --- | --- | --- | --- | --- | --- | --- | --- | --- | --- | --- | --- | --- | --- | --- | --- | --- | --- | --- | --- | --- | --- | --- | --- | --- | --- | --- | --- | --- | --- | --- | --- | --- | --- | --- | --- | --- | --- | --- | --- | --- | --- | --- | --- | --- | --- | --- | --- | --- | --- | --- | --- | --- | --- | --- | --- | --- | --- | --- | --- | --- | --- | --- | --- | --- | --- | --- | --- | --- | --- | --- | --- | --- | --- | --- | --- | --- | --- | --- | --- | --- | --- | --- | --- | --- | --- | --- | --- | --- | --- | --- | --- | --- | --- | --- | --- | --- | --- | --- | --- | --- | --- | --- | --- | --- | --- | --- | --- | --- | --- | --- | --- | --- | --- | --- | --- | --- | --- | --- | --- | --- | --- | --- | --- | --- | --- | --- | --- | --- | --- | --- | --- | --- | --- | --- | --- | --- | --- | --- | --- | --- | --- | --- | --- | --- | --- | --- | --- | --- | --- | --- | --- | --- | --- | --- | --- | --- | --- | --- | --- | --- | --- | --- | --- | --- | --- | --- | --- | --- | --- | --- | --- | --- | --- |

***Table S3 |*** Trait loadings for each trait category on the first four PC axes. Highlighted colors indicate the loading with the greatest absolute value for each trait category, indicating which PC axes this trait most closely maps to. R2 indicates a person’s correlation r^2 value, while the pr(>r) indicates the p-value of the pearson’s correlation. Only those trait categories with significate pearson’s correlations were used in our analysis (excluding pelagic and vertebrate-unknown).

| **Trait Category** | **Dim1** | **Dim2** | **Dim3** | **Dim4** | **r2** | **Pr(>r)** |  |
| --- | --- | --- | --- | --- | --- | --- | --- |
| Diet.Inv | -0.72889 | 0.04167 | -0.6703 | -0.13299 | 0.3469 | 0.001 | *** |
| Diet.Vend | 0.39356 | 0.03779 | -0.46356 | 0.79296 | 0.168 | 0.001 | *** |
| Diet.Vect | 0.48843 | 0.23444 | -0.44586 | 0.71252 | 0.0862 | 0.002 | ** |
| Diet.Vfish | 0.57888 | 0.53957 | -0.25372 | 0.55622 | 0.2369 | 0.001 | *** |
| Diet.Scav | 0.5587 | -0.26573 | -0.3645 | 0.69597 | 0.0737 | 0.008 | ** |
| Diet.Fruit | -0.18726 | -0.32053 | 0.11011 | 0.92199 | 0.2439 | 0.001 | *** |
| Diet.Nect | -0.22782 | 0.533 | -0.59354 | -0.55832 | 0.3513 | 0.001 | *** |
| Diet.Seed | 0.23243 | -0.71693 | 0.51959 | -0.40251 | 0.2387 | 0.001 | *** |
| Diet.PlantO | 0.30902 | 0.17516 | 0.85603 | -0.37556 | 0.8138 | 0.001 | *** |
| ForStrat.watbelowsurf | 0.49156 | 0.74537 | 0.18303 | 0.41145 | 0.1404 | 0.001 | *** |
| ForStrat.wataroundsurf | 0.65444 | 0.6442 | 0.24612 | -0.31007 | 0.5829 | 0.001 | *** |
| ForStrat.ground | 0.54253 | -0.78275 | -0.30091 | -0.04906 | 0.9228 | 0.001 | *** |
| ForStrat.understory | -0.50679 | 0.0334 | -0.06758 | -0.85876 | 0.6346 | 0.001 | *** |
| ForStrat.midhigh | -0.67307 | 0.46574 | 0.36013 | 0.44763 | 0.7397 | 0.001 | *** |
| ForStrat.canopy | -0.29276 | 0.29449 | 0.22037 | 0.88261 | 0.3637 | 0.001 | *** |
| ForStrat.aerial | -0.08517 | 0.61942 | -0.61849 | 0.47596 | 0.1045 | 0.001 | *** |
| BodyMass.Value | 0.66712 | 0.3625 | 0.39471 | 0.51744 | 0.3658 | 0.001 | *** |
| Beak.Width | 0.57338 | 0.28482 | 0.43333 | 0.6343 | 0.494 | 0.001 | *** |
| Beak.Depth | 0.63476 | 0.15269 | 0.28748 | 0.7008 | 0.5428 | 0.001 | *** |
| Beak.Length_Culmen | 0.6686 | 0.41561 | -0.03833 | 0.61544 | 0.39 | 0.001 | *** |
| Hand.wing.Index | 0.42982 | 0.72944 | -0.53123 | -0.03109 | 0.8388 | 0.001 | *** |
|  |  |  |  |  |  |  |  |
| Diet.Vunk | 0.50707 | 0.08046 | 0.16379 | 0.84236 | 0.0116 | 0.536 |  |
| PelagicSpecialist | 0.6339 | 0.46511 | -0.47991 | 0.38927 | 0.0427 | 0.062 |  |
